# Recovery Techniques Enabling Circular Chemistry from Wastewater

**DOI:** 10.3390/molecules27041389

**Published:** 2022-02-18

**Authors:** Vahideh Elhami, Evelyn C. Antunes, Hardy Temmink, Boelo Schuur

**Affiliations:** 1 Sustainable Process Technology Group, Process and Catalysis Cluster, Faculty of Science and Technology, University of Twente, Drienerlolaan 5, 7522 NB Enschede, The Netherlands; v.elhami@utwente.nl (V.E.); e.c.evangelistadasilvaantunes@utwente.nl (E.C.A.); 2 Wetsus—European Centre of Excellence for Sustainable Water Technology, Oostergoweg 9, 8911 MA Leeuwarden, The Netherlands; hardy.temmink@wur.nl; 3 Department of Environmental Technology, Wageningen University, Bornse Weilanden 9, 6708 WG Wageningen, The Netherlands

**Keywords:** extracellular polymeric substances, long-chain dicarboxylic acids, medium-chain carboxylic acids, separation technology, unsaturated fatty acids, volatile fatty acids

## Abstract

In an era where it becomes less and less accepted to just send waste to landfills and release wastewater into the environment without treatment, numerous initiatives are pursued to facilitate chemical production from waste. This includes microbial conversions of waste in digesters, and with this type of approach, a variety of chemicals can be produced. Typical for digestion systems is that the products are present only in (very) dilute amounts. For such productions to be technically and economically interesting to pursue, it is of key importance that effective product recovery strategies are being developed. In this review, we focus on the recovery of biologically produced carboxylic acids, including volatile fatty acids (VFAs), medium-chain carboxylic acids (MCCAs), long-chain dicarboxylic acids (LCDAs) being directly produced by microorganisms, and indirectly produced unsaturated short-chain acids (USCA), as well as polymers. Key recovery techniques for carboxylic acids in solution include liquid-liquid extraction, adsorption, and membrane separations. The route toward USCA is discussed, including their production by thermal treatment of intracellular polyhydroxyalkanoates (PHA) polymers and the downstream separations. Polymers included in this review are extracellular polymeric substances (EPS). Strategies for fractionation of the different fractions of EPS are discussed, aiming at the valorization of both polysaccharides and proteins. It is concluded that several separation strategies have the potential to further develop the wastewater valorization chains.

## 1. Introduction

Until a few centuries ago, mankind obtained the vast majority of their consumer goods either directly from nature or via bioconversions. Examples of bioconversions that have long been established include the production of wine and vinegar. The nineteenth century marked a change because the development of the Bessemer process [1] enabled large-scale production of steel offered opportunities for a tremendous variety of new economic activities, including oil and gas drilling as it is performed today. Resulting from the availability of cheap oil and gas, over the course of the twentieth century, many products that were originally primarily obtained from bio-based sources have found oil- or gas-based synthesis paths. Consider, for example, acetic acid, the vast majority of which is currently produced through carbonylation of methanol [2]. The methanol and CO that are needed in this process are obtained via steam reforming of natural gas. In the past, acetic acid used to be produced via aerobic fermentation but also can be produced through anaerobic fermentation, as will be discussed in a later section. However, the natural gas-based route is cheaper, and for that reason, most processes make use of fossil feedstock. Similarly, mono ethylene glycol that can easily be produced from biological sources is still primarily produced from oil via ethylene and ethylene oxide for economic reasons [3]. Many other examples are available, and our typical economic model is that they are produced, used, and then discarded: the linear economy.

Concerns about our climate and the amount of waste generated were incentives for many initiatives to develop biorefineries, which can be seen as an alternative to oil refineries [4], and circular chemistry approaches. One approach for circular chemistry is through wastewater digestion and valorization. Wastewater digestion to produce biogas has been well-developed, and many smaller-scale commercial installations can be found throughout the world. Although of value for our society when replacing the burning of fossil fuels, the added value of biogas is relatively small, and production of higher added value chemicals from wastewater is a topic in many research and development projects. For example, volatile fatty acids (VFAs) such as acetic acid can be produced [5], which can act as an intermediate platform chemical for a variety of other chemicals [6,7]. In addition, productions of larger carboxylic acids are known, including medium-chain carboxylic acids (MCCAs) [8,9,10,11] and even long-chain dicarboxylic acids (LCDAs), have recently been reported [12]. Next to the direct production of small acid molecules, also indirect acid production through thermal degradation of intracellular polyhydroxyalkanoates (PHAs) is a possible route, yielding unsaturated fatty acids [13,14,15,16]. A final class of acidic molecules we consider in this review is the class of polymers containing acidic functionality, such as alginate. Alginate is an example of an extracellular polymeric substance (EPS), and to obtain alginate, it should be recovered from its wastewater and preferably also fractionated from other species present, including other EPS.

Recovery of valuable products from wastewater is not an easy task, especially not if this has to be performed cost-effectively, because the typical product concentrations are low, mostly below 1 wt%, and in the case of extracellular polymeric products, sometimes significantly below 1 wt%. Molecules inside biomass can be concentrated by concentration of the solid biomass, but recovery of molecules that are solubilized in a highly diluted form in the aqueous phase is not straightforward. Yet, recovery of valuable chemicals can be highly advantageous as it is always accompanied by reduction in the organic load in the waste stream, meaning that treatment and recovery can concurrently occur.

In this review, we focus on the recovery of the four classes of acidic extracellular molecules, and we aim to create an overview of the techniques that can be applied to recover them from the fermentation broth, fractionate them from other constituents, and aim to link the most interesting methods to the molecular properties of the molecules to be recovered. Next to acids that are produced purely biochemically, we also include recovery of unsaturated volatile fatty acids through pyrolysis of (extracted) intracellular polyhydroxyalkanoates (PHA).

The paper is structured on increasing product molecule size, starting with VFAs, followed by MCCAs and LCDAs to proceed to polymers of various categories. After introducing the VFA and MCCA platforms, recovery techniques are discussed, followed by a discussion on LCDA production and recovery, and next, the route toward unsaturated fatty acids via PHAs is discussed. After these four acid routes, the paper proceeds with subsections on the recovery of extracellular alginates and other biopolymers from (fermented) wastewater. A general conclusion section follows the techniques that can be applied for recovery from fermented wastewater.

## 2. Volatile Fatty Acids (VFAs) from Wastewater

**Figure 1:**
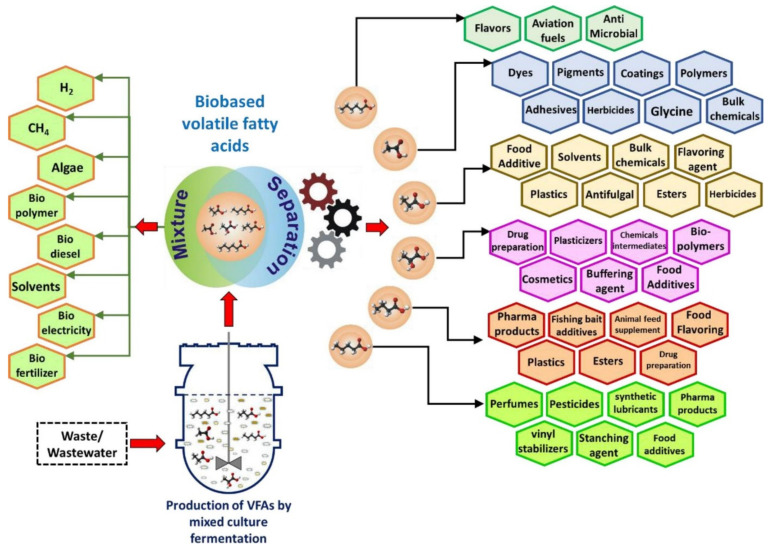
Scope and application of bio-based volatile fatty acids, figure taken from [17].

Short-chain carboxylic acids, VFAs, are used in a wide range of industries producing products such as polymers, food, chemicals, and agriculture [17]. Figure 1 summarizes the application of the VFAs obtained by mixed microbial culture (MMC) using waste/wastewater as a feedstock [5,17,18]. VFAs are used as a building block in manufacturing pesticides, rubber, paint, plastics, and polymers and can also be used as a preservative and flavor in food and beverage industries [18,19]. VFAs are mainly produced via petrochemical pathways that are neither renewable nor sustainable. For example, acetic acid experienced a global market demand of about 15.924 kilotons in 2020, and it is estimated that the demand will increase with a compound annual growth rate (CAGR) of 5% from 2021 until 2026 [20], while 90% of its production is by chemical synthesis from petroleum feedstocks, mainly by methanol carbonylation [2].

The bio-based VFAs can be obtained via the fermentation of a carbon source such as glucose, xylose, sucrose, etc. Fermentation can occur either in pure cultures or in mixed cultures. The former requires sterile conditions to grow a mono-culture containing a single microorganism species fed with pure substrates such as glucose. This biological pathway cannot economically compete with the petrochemical routes due to its low productivity and high manufacturing cost [18]. To improve competitiveness, researchers have focused on providing an inexpensive bio-based alternative that is mainly fermentation of an organic-rich waste/wastewater using MMC [21,22,23,24].

MMC is preferred over the pure cultures as it does not require sterile conditions and enables the application of inexpensive carbon-rich wastes as feedstock, such as whey, biomass, food waste (FW), sewage sludge (SS), and organic fraction of municipal solid waste (OFMSW) [24,25]. SS from municipal wastewater treatment plants (WWTPS) and FW are the most abundant organic wastes with an annual production of about 11 × 10^9^ and 2 × 10^9^ metric tons, respectively [26]. Due to their high organic content, they are the optimal candidates to be used as a carbon source for microorganisms. Besides FW and SS, various solid and liquid wastes have been researched for VFA production, including municipal solid waste, palm and olive oil, wood and paper mill effluent, and dairy wastewater [21,25].

**Figure 2:**
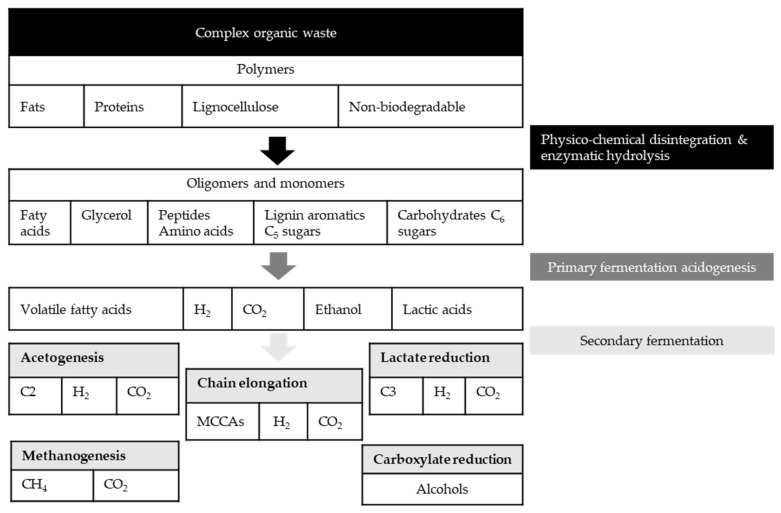
Possible fermentation pathways in mixed microbial culture, reused from [27].

In MMC, the conversion of an organic substrate takes place gradually by different microorganisms, which results in a complicated microbial ecosystem with a versatile metabolic capacity. Figure 2 illustrates the possible fermentation pathways that can occur in MMC. First, the hydrolytic microorganisms hydrolyze the complex biodegradable organics in the waste to their corresponding simple monomers, followed by acidogenic fermentation of these monomers into VFAs, called primary fermentation [25]. Afterward, the VFAs can either be separated from the fermentation broth or undergo several secondary fermentation pathways to produce methane, syngas, alcohol, and MCCAs.

### 2.1. Recovery of the VFA from Fermentation Broth

The acid content of the fermentation broth obtained by waste digestion is reported to be about 1 wt%, and the limited acid content is a result of the limited carbon content of waste/wastewater [28]. From an economic point of view, the separation method must thus be highly selective toward the acids over water. This selectivity can be achieved by affinity separation techniques that enable recovery of a target compound from a relatively complex mixture. The main affinity separation techniques that have been applied to recover VFAs from the fermentation broth include liquid-liquid extraction [29,30,31,32], adsorption [28,33,34], and membrane-assisted separations [33,34,35]. Liquid-liquid extraction of the carboxylic acids from a fermentation broth is well studied [32], with traditional solvents [29], ionic liquids (ILs) [30] and, deep eutectic solvents [31,36]. ILs are preferred due to the higher distribution than traditional organic solvents at highly diluted acid streams. However, the regeneration of ILs is still challenging [6]. Alternatively, membrane-based separations are feasible techniques to concentrate the acids to some extent. These methods can even provide in situ separation of the VFAs from the fermentation broth and prevent the inhibitory impact of the VFAs on the metabolism of the microorganisms and increase the acid production yield [37]. The performance of various membrane types to recover VFAs has been reviewed [38,39,40]. In general, these techniques are potentially able to enhance the recovery of VFAs. However, each of them bears some limitations toward application as a stand-alone economically viable separation technique to recover VFAs. The main drawbacks associated with the various membranes are membrane fouling, high energy demand, and not being selective enough toward VFAs in the complex mixture of the fermentation effluent. Furthermore, the high cost of membrane maintenance and replacement hinders the economy of operation [38]. Bόna et al. [35] applied nanofiltration (NF), reverse osmosis (RO), forward osmosis (FO), and supported liquid membrane (SILM) to recover VFAs from a model fermentation solution. Among them, NF exhibited the highest selectivity between different acids, which might be even further improved by the new type of NF membranes, which are asymmetric polyelectrolyte multilayer (PEM) membranes [41]. Polyelectrolytes (PEs) are polymers with charged repeating units and consequently hydrophilic materials and less prone to foul [42]. As fouling is one of the main challenges associated with hydrophobic conventional membranes, resulting in high operational cost [42], replacement with PEs appears highly interesting. Moreover, integrating a membrane-based separation technology with a conventional recovery method (e.g., liquid-liquid extraction and adsorption) might provide an economic acid recovery approach in which pre-concentration is performed with the membrane separation and fractionation and recovery with either extraction or adsorption.

Adsorption is a technique that can result in high concentration factors [16]. Adsorption is one of the affinity separation methods that have shown great potential to effectively recover VFAs from dilute solutions [28,33,34]. Depending on the physiochemical nature of the adsorbent and fermentation effluent, high selectivity toward VFAs in the complex solutions can be achieved by adsorption.

Since acid recovery using liquid-liquid extraction [32,43] and membrane-based technologies [38,39] have recently been extensively reviewed, the reader is referred to these reviews for an extensive overview of these technologies. However, since the liquid-liquid extraction reviews have appeared in 2018 [43] and 2019 [32], we include here a brief section on the latest developments in recovery of VFAs by liquid-liquid extraction technology, while the main focus of the present review is on adsorption and we evaluate the current adsorbents and their regeneration methods.

#### 2.1.1. Liquid-Liquid Extraction for Recovery of the VFAs

Since the last major review in this field [32], several papers have appeared that report interesting new developments in the field. The relatively young field of hydrophobic deep eutectic solvents (DESs) is developing to find new application areas, and VFA extraction is one of the topics that have been studied in the past few years [36,44,45,46]. Although the use of DESs appears of interest, none of the papers [38,46,47] reports acid distributions that are really excitingly high. The most interesting aspect of changing from traditional solvents to DESs is that by using the DES concept in the formulation of the composite solvent (extractant + diluent), also diluents can be applied that are normally solids, including many bio-based diluents. In this way, bio-based solvents can be developed that have the potential to replace oil-based solvents.

A main challenge for the recovery of carboxylic acids from fermented wastewaters remains that the pH is typically too high for effective extraction because cultures operate best at pH > 5, while under these conditions, extraction is not favored. To extract the neutral acids optimally, the pH should be below the pKa, which for VFAs is around 4.8. Traditional methods for recovering acids from fermentation broths typically involve the addition of sulfuric acid after first adding calcium hydroxide to keep the pH high enough to continue the production. The combination of calcium and sulfate produces gypsum and is not desired for VFA recovery. Ideally, the pH can be swung without producing salts as a byproduct. For such pH swings, two approaches have been reported in the last decade. Expanding the biphasic system with pressurized CO_2_, primarily aiming at a reversible lowering of the pH to enhance the extraction, is the first of the two methods [47]. This approach has shown as an additional interesting benefit, that for some solvents, the extraction performance was improved significantly more than could be expected only on the basis of the anticipated pH swing. In addition, the solvent properties under CO_2_-pressure have improved, leading to a maximum of seven-fold better extraction. The second approach is to use an electrochemical pH swing, and this technique has been demonstrated for succinic acid and itaconic acid [48,49] in combination with recovery by crystallization. When this approach is to be applied for VFAs, an alternative recovery approach needs to be included in the process design, as the VFAs have a too high solubility to be able to crystallize them.

#### 2.1.2. VFAs Recovery via Adsorption-Desorption Technique

Adsorption is a surface-based process involving the adhesion of a compound from a liquid or gas to a surface [18]. The efficiency of an adsorption process depends on the physiochemical properties of both the adsorbate and the adsorbent, as well as on the fluid phase properties. Surface chemistry, pore size, and internal surface area are the relevant characteristics of an adsorbent, which directly influences its capacity. The adsorption potential of a VFA on a specific solid matrix is defined as the equilibrium VFA loading on the resin as a function of the equilibrium aqueous phase concentration, which is expressed in Equation (1):

(1) $$q_{VFA}\left[g_{VFA}/kg_{resin}\right]=f({\left[VFA\right]}_{eq})$$

where **[VFA]_eq_** is the concentration of the **VFA** in the aqueous phase at equilibrium. Depending on the concentration, **q_VFA_** may be in the linear regime [50], but often multiparameter isotherms are applied to describe the loading as a function of the concentration. Higher loadings indicate stronger interaction between the adsorbent and the solute. Various solid matrices have been employed to recover VFAs from a dilute aqueous solution. They can be categorized into three main groups: activated carbon, synthetic polyaromatic resins, and functionalized synthetic polyaromatic resins.

##### Activated Carbon

Activated carbon (AC) is a carbonaceous solid obtained from biomass or coal. ACs are used in various applications due to their high surface area, highly developed pore morphology, and high adsorption capacity [51]. AC has also been applied to recover VFAs from either a fermentation broth or a mimicked broth [17,52,53]. Ahasa Yousef et al. [52] found 42.68 mg g^−1^ adsorption capacity with activated carbon to separate butyric acid from a broth obtained by dark fermentation of food waste containing 6.6 g L^−1^ butyric acid. Recently, a remarkable adsorption behavior was reported for acetic acid, going against the trend with a maximum loading of over 100 mg g^−1^ on activated carbon at equilibrium aqueous concentrations up to 20 g L^−1^, higher than propionic acid and butyric acid loadings [50]. The peculiar result was presented as a decrease in the adsorption capacity of activated carbon at increased acid chain length. We are of the opinion that most likely, the acetic acid results are off for some reason since all other acids in their publication showed the usual trend with increased capacity at increasing acid molar weight, explained by the increasing hydrophobicity. When the recovery of the protonated acids is preferred, the pH of the solution plays a key role in separation. The optimum value of the pH depends on both the isoelectric point (IP) of the adsorbent and pK_a_ of the acid. The IP of activated carbon is 5.48, meaning that its surface is negatively charged at pH > 5.48 and positively charged at lower pH [54]. Meanwhile, the pK_a_ of the acids determines whether it is at its dissociated form at applied pH or not. The pK_a_ value of the acetic, propionic, and butyric acid are 4.75, 4.87, and 4.81, respectively. In order to adsorb the VFAs with non-functionalized AC from aqueous solutions, the pH of those solutions should ideally be between the IP of the AC and the pK_a_ of the corresponding acid.

##### Synthetic Polyaromatic Resins

**Figure 3:**
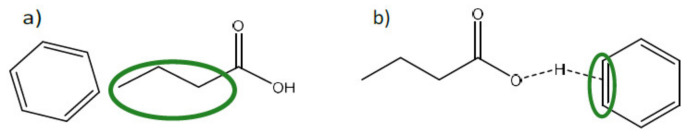
Possible interactions between the aromatic ring of a non-functionalized polystyrene based adsorbent and VFAs, (**a**) purely hydrophobic interactions, and (**b**) hydrogen bond–pi interactions.

The non-functionalized polyaromatic sorbents are able to attract the carboxylic acids by physical interactions such as hydrophobic interaction between the acid hydrocarbon chain and solid matrix and hydrogen bond interaction between their carboxyl groups and the aromatic ring of the adsorbent (see Figure 3) [28]. The former can also be explained by Traube’s rule, describing the crucial rule of acid hydrophobicity in the capacity and selectivity of an adsorbent [53]. Therefore, the longer the VFA carbon chain, the stronger interaction with the resin.

**Figure 4:**
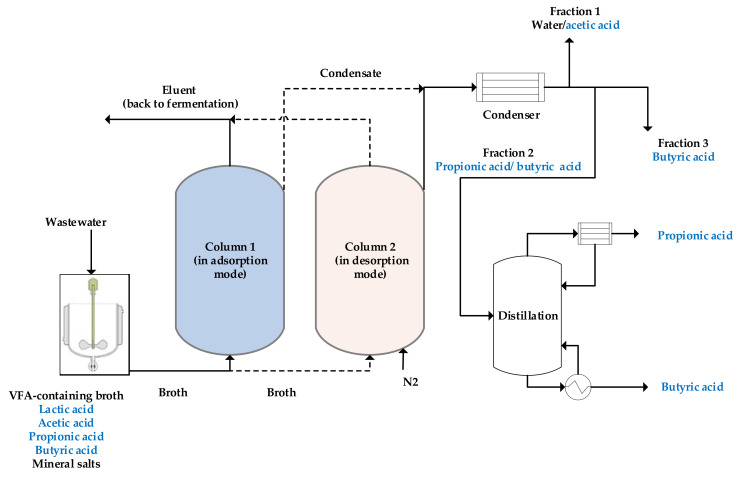
Schematic view of adsorption-thermal desorption process to recover VFAs from a fermentation broth, redrawn from the work of [36] with permission from the American Chemical Society.

Non-functionalized styrene-divinylbenzene-based copolymer with the commercial name of Lewatit VP OC 1064 MD PH has shown a great potential to adsorb the VFAs from a complex mimicked fermentation broth [7,28]. Similarly, it can attract undissociated carboxylic acids via the aforementioned physical interaction mechanisms. A total VFA capacity of 76 g/kg is reported for non-functionalized styrene-divinylbenzene-based adsorbent at an aqueous concentration of only 1 wt% total VFA loading [28]. This adsorbent is gaining more interest as it facilitates selectively recovering the acids in their undissociated form from a complex stream that contains mineral acids and salts as well. Moreover, complete regeneration of the adsorbent is possible via thermal desorption without reducing the capacity of the adsorbent [28]. A schematic view of a complete adsorption-thermal desorption process to recover VFAs from a broth using non-functionalized adsorbent is shown in Figure 4. Reyhanitash et al. [28] found that applying a proper temperature profile based on the boiling point of the corresponding acids enables to not only effectively regenerate the adsorbent but also fractionate the acids during desorption.

#### 2.1.3. Functionalized Synthetic Polyaromatic Resins

##### Anion Exchange Resins

Anion exchange (AEX) resins are widely used to separate carboxylic acid from the fermentation broth. The mechanisms of these resins to attract the acids have been reviewed by Lόpez-Garzόn et al. [55]. Here, we briefly introduce the main mechanisms and will focus more on the comparison between all used adsorbents in terms of processing and including regeneration methods in the next section. AEX resins are polymeric matrices with primary/secondary/tertiary amine or quaternary ammonium functional groups. The adsorption mechanisms of these functionalized polymers depend on several factors such as their basicity, pK_a_ of the acids, and the pH of the fermentation broth. A summary of adsorption characteristics and adsorbents used is presented in Table 1. As can be seen, when a carboxylic acid is the target for weak base sorbents, the dominant adsorption mechanism is H-bonding between the amine group of the resins and the carboxyl group of the acids. Acid-base reaction is also possible when the basicity of the ammonium compound of the resins is higher than the basicity of the conjugate base of the carboxylic acids. The ammonium functional group of the resins offers an opportunity for anion exchange when the basicity of this ammonium salt is higher than the conjugate base of the carboxylic acid.

**Figure 5:**
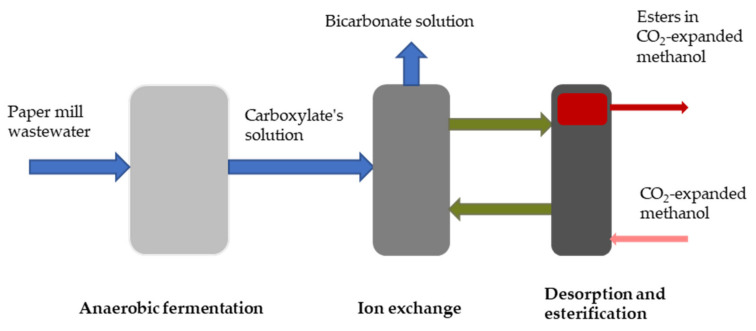
Integrated recovery and esterification of the carboxylate from a fermentation broth by CO_2_-expanded methanol technique and using paper mill wastewater as a feedstock, redrawn from [58].

To achieve effective carboxylic acid production by fermentation, the broth must be titrated with a base to retain the pH neutrality as an acidic environment inhibits the metabolism of the microorganisms [56]. Thus, the pH of the fermentation broth during mixed microbial culture is usually between 5 and 7, which is higher than the pK_a_ of the acids, and as a result, carboxylate anions dominate over undissociated acids in the broth [57]. Hence, separation with an anion exchange resin does not require pH adjustment. However, the regeneration of the resin and recovering the carboxylates in their acid form is not straightforward, requiring an extra agent (e.g., H_2_SO_4_) to protonate them, which results in a stoichiometric amount of salt production [56]. To circumvent this drawback, Cabrera-Rodríguez et al. [56,58] have applied CO_2_-expanded methanol, which generated HCO_3_^−^ by reaction with water, while the proton liberated during this reaction protonated the carboxylates loaded on active sites of the AEX resin. The undissociated carboxylic acids were directly esterified with methanol. By this method, the AEX resin gets regenerated in methylcarbonate/bicarbonate form and the acids are converted to their corresponding methyl ester. In fact, the acids are in the solution with methanol and CO_2_ after regenerating the resin by CO_2_-expanded methanol. Afterward, they can either be recovered from the solution by a separation method (e.g., distillation) or directly undergo another reaction step to produce other chemicals such as esters. By means of this method, Cabrera-Rodríguez et al. [56] could reach a desorption yield of 0.79 mol acetic acid per mol acetate entering the desorption process at 10 bar CO_2_ and ambient temperature using a mimicked fermentation broth. Furthermore, the esterification yield in the combined desorption-esterification is reported to be 1.03 mol methyl acetate per mol acetate at 5 bar CO_2_ and 60 °C. Later on, the feasibility of this integrated recovery and esterification of the carboxylate process using real wastewater was also investigated [58]. The recycled paper industry wastewater has been applied to anaerobically produce carboxylates, followed by recovering the carboxylates by an AEX resin and desorption-esterification by CO_2_-expanded methanol (see Figure 5). The results indicated a successful ester production with the yields ranging from 1.08 ± 0.04 mol methyl acetate per mole of ingoing acetate to 0.57 ± 0.02 mol methyl valerate per mol of ingoing valerate.

#### 2.1.4. Comparative Assessment of Various Desorption Techniques

Various adsorbents have been studied to recover the carboxylic acids from either a synthetic or a real fermentation broth [28,52,55,59,60]. Selecting a proper adsorbent depends on the hydrophobicity and acidity of the adsorbate, adsorbent nature, ionic strength, solubility, presence of the other ions in the broth solution, temperature, and pH [53]. Another important factor that should also be considered is the desired final form of the target molecule. Whether a carboxylate salt or a carboxylic acid is to be obtained should guide the choice for the type of adsorbent and regeneration method. The main methods that have been employed to regenerate the adsorbents are thermal desorption by hot N_2_ [7,28] and washing the loaded adsorbent with a basic aqueous or alcoholic solution [50,59]. In Table 2, a comparison of the advantages and limitations of these regeneration methods for various applied sorbents is given. For example, synthetic polyaromatic resins such as non-functionalized polystyrene divinylbenzene copolymers provide an opportunity to directly recover the carboxylic acids from the fermentation broth at a pH < pK_a_ [7,28]. This adsorbent is able to target the acids over the salts presented in the complex solution of a fermentation broth. The regeneration of this adsorbent is possible via both thermal desorption and washing with a basic solution yielding to collect free acids and carboxylate salts, respectively. Due to the physical interaction between the acids and the adsorbent, the thermal desorption technique enables the recovery of the acids and completely regenerates the adsorbent without damaging its morphology. The main limitation of the aforementioned adsorbent is the adjustment of the pH of the broth prior to the adsorption when the pH of the broth is much higher than the pK_a_ of the acids. The pH of the fermentation broth can be higher than the pK_a_ of the VFAs in which the acids are present in their carboxylate form. Based on industrial broth composition data, however, with a mimicking broth, Reyhanitash et al. [28] reported almost a complete recovery of the acids from non-functionalized polystyrene divinylbenzene base adsorbent using hot N_2_ stripping with the adsorbent reusability of at least four adsorption-desorption cycles. They have proven that applying a proper temperature profile for thermal desorption enables to not only fully recover the acids but also fractionate them during regeneration. A potential drawback for thermal desorption by hot N_2_ stripping forms the energy demand to recover the acids. A techno-economic study is still required to assess this process in terms of energy use for resin regeneration.

On the other hand, the functionalized adsorbents such as AEX resins can also target the dissociated carboxylates by forming the ionic bond between the carboxylates and the cationic functional group of the AEX resins [61]. Therefore, there is no need to adjust the pH of the fermentation broth to protonate the carboxylates. Moreover, ion exchange resins do not require high energy demand, compared to methods such as nanofiltration and electrodialysis [57]. However, the regeneration of these resins involves adding extra chemicals to protonate the carboxylates, which result in the coproduction of stoichiometric amounts of salt. In addition, a further separation technique (e.g., crystallization and distillation) is still needed to separate the carboxylate salts from the eluent. As already mentioned in the previous section, a novel regeneration method has been proposed to recover the adsorbed carboxylates from an AEX resin using CO_2_-expanded alcohol [56]. This technique involves simultaneous regeneration of the resin and protonating the carboxylates, followed by esterification of desorbed carboxylic acids. The avoided production of waste salt is the main advantage of this approach.

## 3. Medium-Chain Carboxylic Acid from Wastewater

Medium-chain carboxylic acids (MCCA) such as hexanoic acid (caproic acid, C6), heptanoic acid (enanthic acid, C7), and octanoic acid (caprylic acid, C8) are the most common MCCAs produced by anaerobic fermentation [62]. They can be used as a biopolymer precursor, as a food additive, as an antimicrobial agent, and as a precursor for bio-fuel production [62]. In comparison to VFAs, MCCAs hold higher commercial value as bio-based fuel due to their lower oxygen to carbon ratio and consequently high energy density [60,62], while their value as intermediate for polymers is expected to be higher than as fuel.

As shown in Figure 2, during anaerobic digestion of an organic waste/wastewater to MCCAs, first, the macromolecules in the biowaste must be converted to small intermediate molecules (e.g., lactic acid, ethanol, and VFAs) through hydrolysis and acidification, followed by microbial chain elongation from VFAs to MCCAs [62,63]. Chain elongation (CE) can be conducted through two routes; Reverse β-oxidation and fatty acid bio-synthesis, which are recently reviewed [27,62]. Reverse β-oxidation is the main CE pathway that requires an electron donor compound to add two carbon atoms to the carbon chain of the carboxylic acids during each elongation step. Ethanol [10], methanol [64], lactic acid [65], carbohydrates [66] have been used as electron-donating agents in reverse β-oxidation to produce MCCAs. The final product spectrum depends on the type of starting substrate and electron donor compound. For instance, odd carbon number VFAs as substrates result in mainly odd carbon number MCCAs using ethanol as an electron donor. Due to the unavoidable oxidation of ethanol to acetate, it is not possible to avoid even carbon number acid production during the odd-CE process [62].

Indeed, waste/wastewater feedstocks can provide an economically attractive source for the production of bio-based VFAs. However, downstream processing of the produced acids remains still challenging due to the complexity of fermentation broth and the low product concentrations. Typically, the downstream processing cost counts up to 30%–40% of total production cost [55]. The concentration of the acids in the stream is a key factor that determines the appropriate separation techniques [67]. Because the bio-based approaches through a waste/wastewater result in highly dilute and relatively complex aqueous acid solution, a robust separation technique is required to obtain concentrated acids [28,68]. Therefore, we will focus on reported separation methods and their competitive assessment in the next sections.

### 3.1. Recovery of Bio-Based Medium-Chain Carboxylic Acids

To achieve a high production rate of MCCAs from organic waste through microbial chain elongation, it is necessary to establish an in situ separation technique and directly remove the acids from the fermentation broth. Because the MCCAs are toxic for the microorganism due to their high hydrophobicity, which causes a disruption in the cell membrane [69], in situ separation of these acids is required to reduce acid production inhibition [70]. Various separation technologies have been applied for in situ recovery of the MCCAs from the fermentation broth, including electrolysis [8], membrane-based liquid-liquid extraction (i.e., pertraction) [9], adsorption with AEX resins [57,71] and electrodialysis/phase separation [11].

#### Membrane-Based Liquid-Liquid Extraction

**Figure 6:**
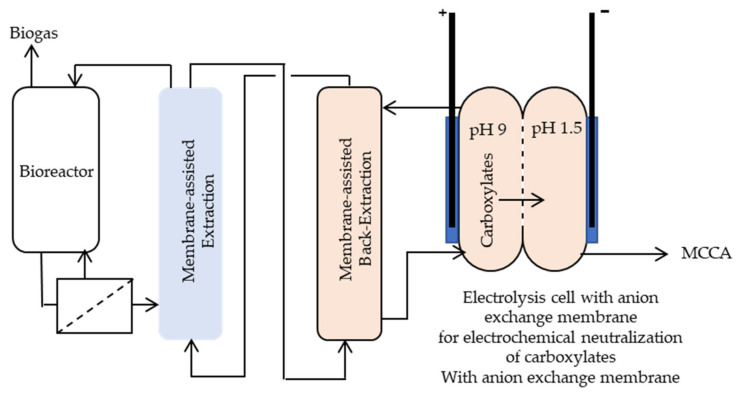
Schematics of the bioreactor and product separation system comprised of membrane-assisted extraction and back-extraction and a membrane electrolysis cell as described in [10].

MCCAs can be extracted into a hydrophobic solvent from a fermentation broth at pH close to pK_a_ of the acids. It can be performed by conventional liquid-liquid extraction in columns. However, the emulsification tendency of two phases is the main limitation for the use of traditional extraction columns. To circumvent this problem, hydrophobic membrane-based liquid-liquid contactors have been introduced, which is referred to as pertraction [72]. By means of these hollow-fiber membranes, it is possible to separate the flow of the organic solvent from aqueous fermentation broth while directly extracting the MCCAs into the organic solvent. Afterward, the acids can be recovered from the solvent either by distillation [73] or by a second pertraction unit [72]. In the second pertraction unit, the acids are recovered from the solvent into a second aqueous stream, which can be an alkaline aqueous solution. Ge et al. [72] have reported 70.3% ± 8.81% n-caproate production yield from yeast-fermentation beer in an anaerobic bioreactor with continuous product extraction by pertraction. The light mineral oil containing 3% tri-n-octylphosphineoxide used as extraction solvent enabled to reach 93% n-caproic acid extraction efficiency. The backward extraction was performed using a 0.5 M borate aqueous phase poised at pH 9. To collect the MCCAs in oil form from a concentrated alkaline solution, the mixture must be acidified to below the pK_a_ of the acids to promote phase separation due to the low solubility of MCCAs. Such phase splitting in which an oily phase is generated is called oiling out [74]. There are two approaches to perform the acidification being either chemically, by adding an acid, or electrochemically, by generating protons via water electrolysis. Hence, membrane electrolysis (ME) enables to extract and acidify the MCCAs without dosing chemicals [10]. In ME, the pH gradient between two chambers allows transferring carboxylates into their acid form without chemical addition. A schematic view of the integrated process of pertraction combined with ME to recover MCCAs is shown in Figure 6. By means of this technique, Xu et al. [10] could recover MCCAs from the fermentation broth with a purity of around 90%.

Similarly, 73% n-caproic acid extraction efficiency was achieved by pertraction, followed by electrochemical phase separation using ME in the work of Carvajal-Arroyo et al. [8], which describes the up-scaling of the previously suggested process by Xu et al. [10]. They have found that ME facilitates harvesting MCCAs in an oil phase after pertraction at the cost of 0.82 € Kg^−1^ oil (assuming an electricity cost of 0.12 € kWh^−1^) in a non-optimized system. However, ME without pertraction is not able to separate the MCCAs.

Later on, an electrodialysis/phase separation cell (EP/DS) was developed to evaluate whether it can act as a stand-alone extraction and separation unit and possibly reduce the capital and operating costs [11]. EP/DS is an electrodialysis system with five chambers that combines two concentrating steps with an internal recirculation loop, promoting phase separation. EP/DS in series with pertraction showed electric power use of 1.05 kWh kg^−1^ MCCA oil, which is 10 times lower than ME in series with pertraction. When EP/DS was applied as a stand-alone unit to directly separate the acids from fermentation broth, the electric power use increased due to the lower acid concentration in the fermentation broth compared to pertraction broth. However, it was possible to selectively extract MCCAs from the bioreactor broth without the need for an additional pertraction step. Overall, it was concluded that a detailed techno-economic analysis is needed to determine whether or not the ED/PS must be combined with pertraction to economically separate MCCA oil from the chain elongation bioreactor. The most recent study evaluated the application of the submerged hollow-fiber membranes for selective MCCA recovery by pertraction, which may offer a further reduction in the capital cost, footprint, and energy usage [9]. These internal membranes resulted in a 3 to 4.7 times higher average extraction rate than the traditional external pertraction loop. Moreover, circulating the broth with an upflow velocity of 7.6 m h^−1^ was sufficient to prevent membrane fouling. However, a further increase in the velocity resulted in a reduction in MCCAs concentration in the broth, and consequently, more methane production by the methanogenesis was favored, showing the importance of proper membrane surface selection and process control. With a properly controlled process, an optimum operating condition to maximize MCCA extraction efficiency and minimize methanogenesis can be achieved.

### 3.2. Adsorption Using Anion Exchange Resins

Next to membrane-based liquid-liquid extraction, the application of AEX resin to adsorb the MCCAs from fermentation broth has also been investigated [61,74,75]. A preliminary study by Rebecchi et al. [59] in batch scale illustrated the highest affinity and selectivity of Amberlyst A21 (a tertiary amine AEX resin) toward MCCAs in the complex carboxylic acids mixture obtained by acidogenic digestion of grape pomaces. A relevant contribution of hydrophobic interactions between the carbon chain of the acids and the aromatic ring of the resin was shown. In terms of desorption, basified ethanol was able to completely regenerate the resin. Similarly, Yu et al. [71] investigated the potential of other AEX resins for in situ separation of caproate from the fermentation of anaerobic mixed culture. A trimethylammonium-functionalized polystyrene AEX resin with the commercial name of D201 (from Frotec) resulted in the highest adsorption capacity of 62 mg g^−1^ of the resin. To evaluate the impact of this in situ separation technique on the production rate of caproate, the fermentation was performed with and without coupling the bioreactor to the separation unit. It was found that combining an adsorption step with the fermentation reactor could enhance caproate production rate from 0.60 ± 0.01 g L^−1^ d^−1^ to 2.03 ± 0.02 g L^−1^ d^−1^. Furthermore, this resin showed suitable reusability for at least eight adsorption-desorption cycles using 1.5 M NaOH solution as an eluent to recover the adsorbed acids. Recently, Fernando-Foncillas et al. [57] investigated the performance of the CO_2_-expanded alcohol technique to desorb the loaded MCCAs on AEX resins. This technique was able to concentrate hexanoate and heptanoate up to 4.6 and 20.7 times, respectively.

In comparison to membrane-based extraction and electrodialysis, adsorption with ion exchange resins reduces the costs due to the avoided membrane fouling and corresponding high energy demand [57]. Moreover, it might be possible to concentrate the MCCAs above their solubility using a proper resin and regeneration eluent. Consequently, the final recovery of the acids can be easily achieved via phase separation, which might lower the costs associated with the eluent recovery to a great extent.

### 3.3. Considerations on MCCA Recovery Techniques

Based on the above-described processes that have been reported for MCCA recovery, several techniques have been shown suitable for this class of acids. Considering their natural boiling points, vacuum distillation can be used as the final step in a separation train comprised of either liquid extraction or non-functionalized resin adsorption. The benefit of distillation is that pure products may be obtained through thermal fractionation, while in comparison with oiling out, a lot more energy is required. Combining oiling out with a membrane separation has potential as it does not require a large heat duty; however, when multiple MCCAs are present in a complex broth, it does not offer fractionation opportunity, and when fractionation is aimed for, this should be realized in either the pertraction stage or in the electrodialysis step when applied. Alternatively, nanofiltration-based approaches [42] could be considered for the MCCAs in combination with oiling out. After a concentration step by the membrane, leading to an oversaturated solution of the poorly soluble MCCAs, an oiling-out recovery may be applied.

## 4. Long-Chain Dicarboxylic Acids from Wastewater

LCDAs are used in various industries such as in the production of powder coatings and grease, in the cosmetic industry as an emulsifier, waxing agent in lubricants, and they are also used in the production of polyesters and polyamides, making use of their double functionality [12,76]. The global market size of C18 dicarboxylic acid is estimated to reach USD 9.4 billion by 2024 [12].

The mechanisms for the biological production of LCDAs have been fully reviewed by Werner et al. [77] and Huf et al. [78]. Briefly, it involves the conversion of hydrophobic compounds such as fatty acids originated from vegetable oils to LCDAs by oleaginous yeast strains. It is a multistep process, starting with taking up the hydrophobic substrates, followed by primary oxidation of the alkanes to fatty acids. Afterward, the fatty acids break down by β-oxidation and then be converted to LCDAs via ω-oxidation. The produced LCDAs naturally undergo further degradation via β-oxidation and shorten the fatty acid chain by yeast. It was found that blocking β-oxidation by genetic modification is essential to enhance the LCDAs biological production [77]. Although some of these engineered yeast strains are commercially available, it is still needed to optimize the cost of biological LCDAs production pathway to compete with fossil-based approaches. For example, this yeast might be fed with a waste/wastewater containing fat instead of pure substrates. Next to the fat content, the non-toxicity of the waste composition for the yeast is crucial. Buathong et al. [75,79] used coconut milk factory wastewater to produce 1,12-dodecanedioic acid (1,12-DDA) using Saccharomyces cerevisiae strain. Because the main fatty acid present in coconut milk factory wastewater is lauric acid (approximately 45%–53%), this can be converted to 12-hydroxydodecanoic acid (12-HDDA) and 1,12-DDA via omega oxidation (ω-oxidation) involving the hydroxylation of the terminal alkyl carbon of a fatty acids to an alcohol, followed by further oxidation to a dicarboxylic acid. At the optimum operation condition (30 °C and pH 5), a yield of 60 and 38 µg L^−1^ was achieved for 12-HDDA and 1,12-DDA, respectively, using coconut milk factory wastewater with 10 g L^−1^ yeast extract and 20 g L^−1^ peptone [75]. Moreover, sugar-containing waste/wastewater would be even more economical as this might not require extra glucose for cell growth. Recently, Bauwelinck et al. [12] applied a chocolate industry side stream as a feedstock for a commercially available oleaginous yeast named C.tropicalis ATCC20962. They could convert 47.5% of the fats present in chocolate water to LCDA. However, it was not achievable without adding extra glucose to the bioreactor as the majority of the sugars in chocolate wastewater could not be metabolized by applied yeast. Their study illustrates the possible potential of some waste/wastewater to be employed for biological LCDAs production. However, more research is needed to optimize these processes to be economically competitive with their petrochemical production routes.

The separation of LCDAs from the fermentation broth depends on the final composition and properties of the broth. Indeed, their separation from an aqueous solution is easier than MCCAs due to their high hydrophobicity and consequently low solubility, which results in their spontaneous precipitation. Alternatively, production in a two-liquid system with in situ extraction into an apolar phase may be pursued. In an early paper by Woodley and co-workers, hexane extraction has proven successful for the isolation of LCDAs [76]. Although the field of LCDA recovery is still highly uninvestigated, the strategies applied for MCCAs based on oiling out after membrane concentration or back-extraction do appear very suitable for LCDAs too. Distillation, on the other hand, will not be a suitable approach due to the high boiling points.

## 5. Unsaturated Short-Chain Carboxylic Acids from Wastewater

### 5.1. Crotonic Acid

Crotonic acid, or trans-2-butenoic acid, is mainly used in the synthesis of copolymers. Among them, crotonic acid-vinyl acetate is the main copolymer, with trade names, Vinnapas, Mowilith, and Vinac [80]. They are commonly used in cosmetic and hair styling products. Apart from this, crotonic acid finds further applications in industries such as coatings, paint, textile, binders, adhesives, flocculants, ceramics, and agrochemicals [16]. Despite the wide range of applications for crotonic acid, its commercial production is limited due to its complex production pathway from fossil oil, while specifically for this unsaturated acid, synthesis via biological pathways appears to be interesting [16,81]. Therefore, in the current study, crotonic acid is selected to be reviewed to point out the possible renewable production routes.

On an industrial scale, crotonic acid is currently produced in a non-renewable pathway from fossil oil, requiring many synthesis steps, which is a major drawback in the production process. It begins with the ethylene production by naphta cracking, followed by the oxidation of ethylene into acetaldehyde, aldol condensation of acetaldehyde into acetaldol, dehydration of acetaldol into crotonaldehyde, and lastly, oxidation of crotonaldehyde into crotonic acid [16,82,83]. After all these steps, the current yield of crotonic acid is only 30%. Further purification involves fractional distillation and crystallization from water and causes product loss. In addition, highly contaminated effluent is formed during the crystallization step [84].

Other, not yet industrial-scale production pathways of crotonic acid include isomerization of vinylacetic acid with sulfuric acid, photochemical oxidation or oxidative irradiation of crotonaldehyde with ultrasound, dehydration of 2-hydroxybutanoic acid, the condensation of acetaldehyde and malonic acid with pyridine as catalyst (85% yield), by the oxycarbonylation of propene with transition metal complex catalysts, the reaction of acetic anhydride with acetaldehyde with basic aluminum acetate as a catalyst, the carbonylation of propylene oxide, the oxidation of butene with a heteropolymolybdic acid-containing catalyst system, and the carbonylation of allyl alcohol in a two-phase system with a nickel-containing phase-transfer catalyst or a palladium catalyst [80]. All the aforementioned pathways are from non-renewable sources, and the production costs of crotonic acid are high.

An alternative option would be to produce crotonic acid from a biological source. Several bacterial species (*Ralstonia eutropha*, *Escherichia coli*, *Corynebacterium glutamicum,* and *Clostridium acetobutilicum*) are capable of producing crotonic acid [16,81]. However, the quantitative yield and purity of the crotonic acid were not reported, and a proper separation method is still needed to recover the crotonic acid from the fermentation broth.

Mamat et al. [16] proposed a different bio-based method for crotonic acid production, in which first the biopolymer poly(3-hydroxybutyrate) (PHB) is obtained intracellularly in the fermentation broth of *Cupriavidus necactor*, after which crotonic acid is obtained by pyrolysis of PHB. They found a yield of 63% crotonic acid, which is 30% higher than the conventional petrochemical route. A variety in approaches toward crotonic acid from biomass is possible, not only including PHB but also other polymers. In addition, direct pyrolysis and extraction and purification prior to pyrolysis are processing variations to be considered. In the next subsections, the production of the bio-based polymers and their conversion into crotonic acid will be discussed in more detail.

### 5.2. Polyhydroxyalkanoates Production from Waste/Wastewater

**Figure 7:**
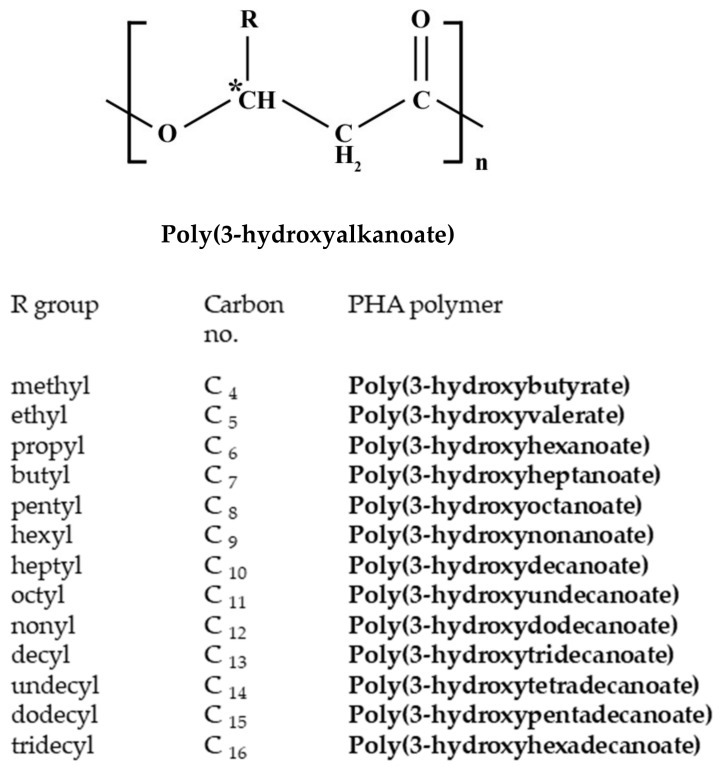
General structure of various PHAs, reproduced from [87].

PHAs are bio-polyesters that function as an energy reserve, ensuring the long-term survival of the bacteria during nutrient-scarce conditions [85,86]. As depicted in Figure 7, PHAs consist of (R)-hydroxy fatty acids ranging between 600 and 35,000 units in length, where R is typically an unsaturated alkyl group [87]. Depending on the total carbon atoms within the monomer, they can be categorized into three groups. The first group is formed by the short-chain length PHA (scl-PHA), consisting of 3–5 carbon atoms such as C_4_ poly(3-hydroxybutyrate) (PHB)and C_5_ poly(3-hydroxyvalerate) (PHV). The second group contains medium-chain length PHA (mcl-PHA), which have between 6 and 14 carbon atoms, and in the third group, the long-chain length PHA (lcl-PHA) are present, which have more than 15 carbon atoms [82].

So far, over 150 types of PHA monomers have been discovered. PHB is an scl-PHA, and the most common type of PHA, discovered by Lemoigne in 1926, while he observed the granules inside a Gram-positive bacterium [83,88].

**Figure 8:**
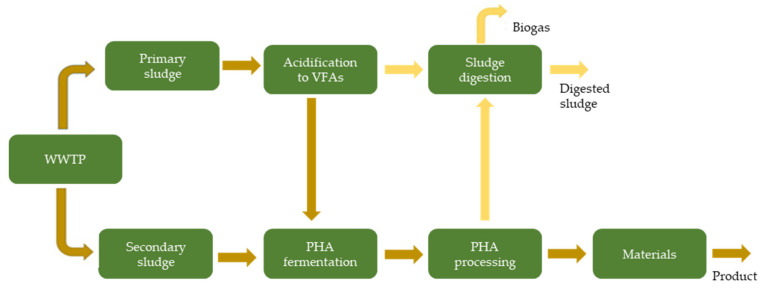
Production process of PHA via a wastewater treatment plant (WWTP) taken from [92].

The PHAs can be produced by either chemical synthesis, use of transgenic plant cells, or by bacterial fermentation [89]. PHAs produced via bacterial fermentation through MMC is preferred over pure cultures as it reduces the fermentation cost because it is not required to pre-treat the substrate, and sterilization is also not necessary. Especially when the pyrolysis products are desired, high-cost substrates and processing appear not necessary, as the polymers are pyrolyzed anyhow. These benefits make MMC an attractive route for PHA production, extending the possibility of using inexpensive carbon sources such as municipal solid waste and industrial wastewater [90]. Synthesis through an MMC such as a wastewater treatment plant (WWTP) occurs in three stages, as depicted in Figure 8. First, the primary sludge undergoes acidogenic fermentation to produce VFAs using either an anaerobic-aerobic (AN/AE) process, an aerobic dynamic feeding, or through fed-batch systems [90,91]. Afterward, the secondary sludge, which acts as an incubator for microorganisms, is enriched with these VFAs, followed by accumulated growth of PHAs in the secondary sludge and finally polymer recovery in the downstream process where it can then be used in different materials [88].

During the fermentation process, VFAs with an even number of carbons will produce more PHB, while those with an odd number will produce a copolymer poly(hydroxybutyrate-co-hydroxyvalerate) (PHBV) with varying hydroxyvalerate (HV) ratios [93], and the PHBVs bioaccumulate within the intracellular lipid as granules ranging between 0.2 and 0.5 µm [82].

Various separation techniques have been proposed to extract the PHA granules from the cells [94,95,96,97]. For extensive reviews on the production and recovery of PHA from MMC, the following papers are suggested reading: [31,98,99,100,101,102,103]. Here, we only focus on the production of crotonic acid from PHA as a downstream process to valorize the bio-based PHBV that is produced from wastewater and does not meet polymer market quality.

Due to the daily variation in the composition of the waste/wastewater, the produced PHBV copolymer varies in its monomer composition, which can influence the properties of the polymer, such as melting point and crystallinity. Clearly, a polymer with variable properties per batch cannot find a broad application window in the polymer market. Instead, it can be depolymerized toward other value-added chemicals such as crotonic acid, which is further discussed in the next section.

### 5.3. Crotonic Acid Synthesis by PHB/PHBV Pyrolysis

Depolymerization of PHA can be achieved by hydrolysis, alcoholysis, and thermolysis, resulting in 2-hydroxyalkanoic acids, 2-hydroxyalkanoic esters, and 2-alkenoic acids, respectively. So far, production of trans-crotonic acid (referred to as crotonic acid) and cis-crotonic acid (referred to as isocrotonic acid) [14,16,104,105,106,107], methyl acrylate [108], methyl crotonate [108,109], propylene [110,111,112], 3-hydroxybutyric acid [16,104], methyl 3-hydroxybutanoate [113], cyclic and linear oligomers [114], and hydrocarbon oil [115] from PHB depolymerization have been reported. PHBV is the main biopolymer that can be obtained by MMC, and during the thermal breakdown of PHBV, crotonic acid and 2-pentenoic acid are the main products [116]. After decomposition of PHB/PHBV, recovery of the unsaturated acids from the pyrolysis mixture is required.

Pyrolysis is a thermal degradation process of organic materials in an inert (i.e., oxygen free) atmosphere [117]. An inert atmosphere is required to prevent thermo-oxidative reactions [118]. By subjecting organic material to high temperatures, the molecules decompose into smaller molecules [117]. The pyrolysis products can be categorized into three categories: char, non-condensable gasses, and condensable gasses [117]. The yields of the products formed depend on many factors, such as the raw material used, temperature, reaction time, heating rate, and cooling rate.

**Figure 9:**
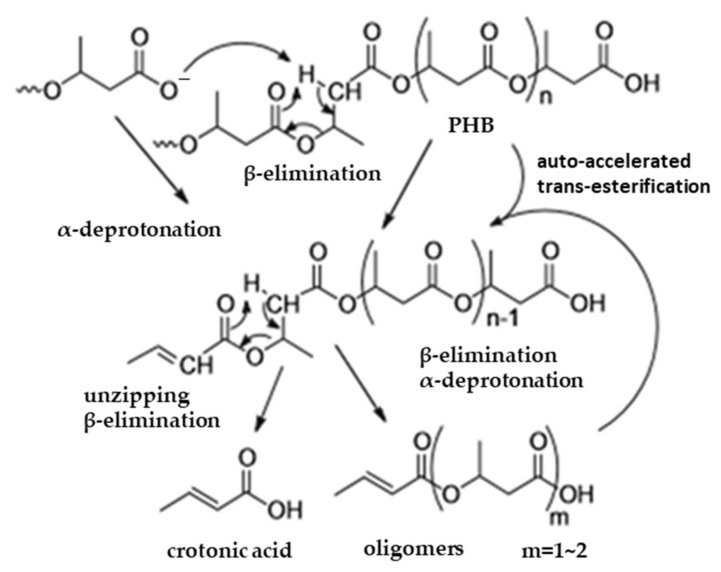
Thermal degradation of PHB toward crotonic acid, by H. Ariffin et al., reproduced with permission from [13]; published by Elsevier, 2008.

Several studies were performed on the thermal degradation mechanism of PHB/PHBV, in which different reaction paths were considered. Overall, it was concluded that a beta elimination, followed by an unzipping beta elimination at the crotonyl chain end, is the dominant reaction path [13,15]. This path is schematically shown in Figure 9. The degradation rate of PHB/PHBV depends on the reactivity of the beta hydrogen. The relative acidity of the acid formed with the removed hydrogen atom determines the reactivity of the beta elimination. The carbonyl group that nucleophilically attacks the beta hydrogen can be interpreted as the Lewis base.

Several heterogeneous catalysts have been studied to enhance crotonic acid production through pyrolysis of PHB(V) such as Mg(OH)_2_, Ca^2+^, Mg^2+^, and Na^+^ [14,119,120,121]. It was found that some metal ions act as electrophiles and thereby increase the reaction rate. The metal ions as a Lewis acid interact with the carbonyl group, thereby enhancing the reactivity of the beta hydrogen toward the carbonyl group. Next to the heterogeneous catalysts, acetic acid was also investigated as a homogeneous catalyst [118]. Liquid catalysts with Lewis acidic behavior similar to the solid catalysts and their ability to dissolve the polymer or loosen up the structure may catalyze the thermal degradation of PHB/PHBV even better.

Table 3 summarizes the PHB/PHBV pyrolysis experiments that have been reported. As can be seen, crotonic acid production yield without using a catalyst ranges from 57 to 65 wt%. While the catalyzed pyrolysis leads to 83 wt% crotonic acid yield with a purity of 97.7% [14]. The produced side-products are terminally unsaturated oligomers and isocrotonic acid. 2-pentenoic acids are also formed from polymers that contain an HV monomer. Secondary products can also be formed by the further decomposition of the initial products, including: propylene, CO_2,_ and acetaldehyde [118]. The formation of crotonic acid is favored over the formation of isocrotonic acid as the trans form is more stable, which is beneficial for the synthesis of copolymers with vinyl acetate, which requires crotonic acid and not isocrotonic acid. Aiming at the production of crotonic acid by thermal treatment of PHB/PHBV, it should be considered that when temperatures exceed 100 °C largely, isocrotonic acid can be formed through an isomerization reaction [122]. The use of catalysts such MgO, Mg(OH)_2_ [14], and Ca^2+^ [121] that lead to selective production of crotonic acid is desired because various authors reported that thermal degradation of PHB/PHBV occurs at a temperature range of 240–310 °C (see Table 3). With the use of catalysts, about 20–40 °C reduction was observed in the degradation temperature of PHB/PHBV [14,121].

### 5.4. Purification of the Crotonic Acid Obtained by PHB/PHBV Pyrolysis

**Figure 10:**
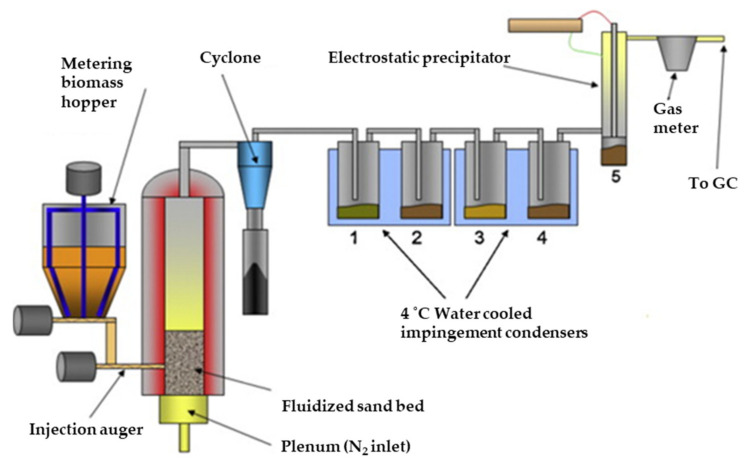
Schematic view of fluidized bed PHB pyrolysis process. The figure is reproduced with permission from [105], published by Elsevier, 2014.

Although the synthesis of crotonic acid via PHB/PHBV thermal decomposition haves been examined over the last decades, its separation from the complex pyrolysis mixtures has not been that well studied yet. Mullen et al. [105] have developed a fluidized bed set-up on a pilot scale to pyrolyze PHB/switchgrass blend and produce crotonic acid-enriched bio-oil. They applied multistep condensation using water-cooled (4 °C) condensers connected to an electrostatic precipitator for in situ fractionation of the pyrolyzates and production of bio-oil enriched with crotonic acid. The schematic view of this process is shown in Figure 10. The use of an electrostatic precipitator can be explained considering the melting point of crotonic acid, being 70 °C. Cooling of a vapor stream containing crotonic acid with cooling water of 4 °C will not only result in condensation but also in partial precipitation. The presence of precipitated crotonic acid in aerosols that escaped the condensers through the vapor phase was confirmed experimentally [105]. The maximum crotonic acid yield of 45% was obtained from a mixture of 10% PHB and 90% switchgrass using fine PHB particles at 375 °C. However, significant fractionation of crotonic acid from the total pyrolysis liquid was not achieved with this multistep separation procedure. The concentration of crotonic acid reduced from 11.2 to 8.7 wt% over the series of condensers without following a pattern based on either water solubility or vapor temperature.

Recently, Parodi et al. [107] developed a novel thermolytic distillation process enabling pyrolysis of PHB and PHB-enriched bacteria at 170 °C under reduced pressure of 150 mbar to yield crotonic acid. In thermolytic distillation at mild conditions (170 °C and 150 mbar), the pyrolysis of PHB occurs simultaneously with the separation of volatile pyrolyzates such as crotonic acid. The integrated product removal at such mild conditions limits the isomerization of crotonic acid to isocrotonic acid as the isomerization reaction occurs mostly at high temperatures. By means of this integrated technique, they could recover crotonic acid with a yield of 58% and a purity of 92% from MMC-based biomass containing 30 wt% PHB on a dry basis. Indeed, pyrolysis of MMC-based PHB/PHB-enriched biomass using this novel method can increase both the yield and purity of the crotonic acid. However, the vapor stream will still also contain 2-pentenoic acids, originating from the HV monomer. Therefore, a next purification step is required to separate these carboxylic acids.

Table 4 represents the molecular properties of the mentioned carboxylic acid. The difference in their boiling and melting point is sufficient to consider distillation and crystallization as a separation approach, although careful analysis of the vapor-liquid equilibria and solid-liquid equilibria should be studied. For affinity-based separation techniques (e.g., extractive distillation, liquid-liquid extraction, and adsorption) to work, their similar molecular properties such as pK_a_ and both having a double C=C bond are expected to limit the operational window. For extractive distillation, the difference in pK_a_ may either be too small or just enough [123]. The difference in hydrocarbon backbone length may result in a feasible difference in distribution over a polar phase and an apolar phase, suggesting that liquid-liquid extraction might offer opportunities for fractionation.

## 6. Extracellular Polymeric Substances (EPS)

### 6.1. Production of EPS from Wastewater

In this subsection, first, the production of biopolymers from wastewater is considered, and in the next subsection, the methods that can be applied to recover the biopolymers are discussed.

Biopolymers are the building blocks of nature as they consist of macromolecules formed during the life cycle of all animals, plants, and microorganisms. Based on their repeating unit, these natural polymers can be classified as proteins (composed of amino acid units), genetic material (polymers of nucleic acids), polysaccharides (composed of sugar or sugar acids), PHAs, and polyphenols [130].

Besides being inherently available in nature, biopolymers are also found in wastewater. For example, wastewater from the dairy industry is a source of biopolymers. It is estimated that 2.5 to 3.0 L of wastewater is generated for every liter of processed milk [131,132,133], typically rich in casein, a milk protein. Casein is typically found in concentrations up to 0.4 g/L, and it is considered one of the main contributors to the organic load in dairy wastewaters [134]. Poultry processing wastewater is another source of protein, with a protein content of about 35% [135,136]. Soy whey wastewater is a byproduct generated during the production of soybean products and contains approximately 1% carbohydrates (mostly comprised of galactose, glucose, and fructose units) and 0.1%–0.8% of protein [137,138]. Significant amounts of polyphenols are found in olive mill wastewater and can be recovered by membrane processes, as reported by Garcia-Castello et al. [139]. The authors proposed a process involving microfiltration (MF), followed by a concentration step using nanofiltration (NF) and osmotic distillation (OD). MF allowed the recovery of 78% of the initial polyphenol content and, after the concentration step, a stream containing 0.5 g/L of polyphenols was obtained. As shown, the concentration of biopolymers in wastewater is often relatively low; therefore, the direct recovery of the biopolymers from wastewater is not economically feasible.

Alternatively, wastewater can be used as raw material for the production of biopolymers by a vast array of biotechnological processes [130,140]. As the opposite to plant-based biopolymers, the production of microbial biopolymers is not easily affected by changes in regional and climatic conditions and presents high growth rates [141]. Microbial biopolymers may occur intracellularly or extracellularly. In this section, only extracellular polymer (EPS) is discussed, and since PHAs are an example of intracellular biopolymer accumulated in the cytoplasm of cells, they will not be further considered.

EPS is a general term for a series of macromolecules, namely polysaccharides, proteins, and glycoproteins produced by bacteria, fungi, and algae [142,143]. Nucleic acids can also be found in EPS as a result of microbial cells lyse rather than actively excreted by microorganisms. The main advantage of recovering valuable polymers from EPS instead of from intracellular polymers is that cell disruption, an energy-intensive process, is not required, so separation of the biopolymer from the biomass is relatively simpler [144].

Based on their association with the cells, EPS can be classified into two general groups: bound EPS (attached to the cells) and soluble EPS (solubilized into the culture aqueous media). These fractions can be separated by centrifugation, where the precipitate represents bound EPS, and the supernatant consists of soluble EPS [142,145]. For bound EPS, solubilization is needed prior to further purification steps. Physical and chemical methods have been reported as effective ways to solubilize bound EPS [142,145]. Cation exchange resin and heating are examples of physical methods to dissolve EPS in water. Cationic resins work by disrupting the interactions between divalent cations and negatively charged functional groups of EPS macromolecules, which are responsible for the formation of bound EPS [146,147]. Chemical methods include the use of aqueous solutions of sodium carbonate, ethylenediaminetetraacetic acid (EDTA), or formamide to dissolve EPS [146,148]. As an emerging solvent class, ionic liquids (ILs) are also solvents that have been shown to be effective in dissolving bound EPS, as demonstrated by Boleij et al. [149]. The chemical methods also work by disrupting the interaction between the biopolymer chains and the interactions with the cells, enhancing, thus, the EPS solubilization into the aqueous media [144]. Since there is a considerable amount of review papers about recovery techniques for a bound fraction of EPS [150,151], recovery techniques for this type of biopolymer are not included in the scope of this paper, being a soluble fraction of EPS, the target fraction of this review.

When it comes to the production of extracellular microbial polymers, single-culture systems are already well studied [152,153,154] and carried out on an industrial scale, for example, for cellulose, pullulan, xanthan gum, and hyaluronic acid [155,156]. On the other hand, literature shows few examples of the production of EPS by mixed cultures.

Mixed cultures present a higher yield of EPS than single cultures due to the symbiotic relationship among the microorganism in this type of culture, which leads to better conversion of the substrate [144]. In addition, the cost of production of EPS by mixed cultures is lower as they do not require sterile conditions for growth or expensive carbon sources. Wastewater is an example of an inexpensive carbon source for mixed cultures. The main advantage of producing EPS from wastewater is that it allows obtaining value-added biopolymers while reducing the organic load of the effluent. Significant amounts of microbial biopolymer can be obtained by employing wastewater with certain characteristics. For instance, diverse authors have shown that the type of substrate present in the wastewater affects the production and composition of EPS. Sponza [141] employed a series of wastewater and found out that winery and municipal wastewater led to the highest production amount of EPS, mostly composed of proteins, among the wastewaters investigated. Ajao et al. [142] demonstrated that significant production of EPS can be achieved using glycerol and ethanol as substrate. In this case, it was possible to convert up to 60% of the carbon source into biopolymers, yielding a solution of 1–2 g/L of soluble EPS. Li and Yang [143] found that using glucose as a substrate led to higher EPS production than using acetate. Another relevant characteristic of wastewater is the carbon to nitrogen (C/N) ratio [144]. The authors of [157,158,159,160] reported that a low C/N ratio leads to EPS with a high protein/polysaccharide ratio, whereas a high C/N ratio produces a higher EPS amount, which is predominately composed of polysaccharide. 

Wastewater can thus play a relevant role in promoting circular chemistry by representing an inexpensive carbon source for the production of value-added biopolymers. However, the implementation of this concept is still hindered by the lack of efficient and cost-effective recovery techniques [141,161]. In the next sessions, recovery techniques for recovery of biopolymers are reviewed, and applicability on recovery of EPS from mixed cultures is discussed.

### 6.2. Concentration and Fractionation Techniques for EPS

The downstream processing of EPS from fermented wastewater usually involves three main steps, namely extraction, fractionation, and concentration. The extraction steps aim at separating biopolymers from the original matrix. Fractionation separates the different types of biopolymers, and concentration separates the biopolymer from the solvent. In the following subsections, recovery techniques for the fractionation and concentration steps are discussed.

#### 6.2.1. Solvent Precipitation

Solvent precipitation is the most commonly used technique to recover biopolymers on an industrial scale [162,163,164]. The aim of this technique is to concentrate and fractionate biopolymers, often present in diluted solutions. The separation occurs by the addition of an agent (precipitant), which is able to change the nature of the solvent (i.e., polarity or ionic strength). As a result of the changed solvent properties, the biopolymers aggregate, and when they are large enough, they can be separated by settling or centrifugation at reasonably low g forces.

The precipitation of polysaccharides occurs by the addition of organic solvents such as acetone, methanol, ethanol, or isopropanol [165,166] and surfactants such as cetyltrimethylammonium bromide (CTAB) [167]. These organic solvents act as dehydrating agents due to their affinity for water molecules, and it reduces the solubility of polysaccharides, a strongly polar macromolecule, in the media. The performance of this separation method depends on the type of organic solvent used, its concentration, and temperature. According to Bahl et al. [168], significative precipitation of polysaccharides occurs only at a solvent concentration above 40% (*v*/*v*).

The precipitation of proteins is usually induced by ammonium sulfate (NH_4_(SO_4_)_2_) or trichloroacetic acid (TCA) as a precipitant [169,170]. Little is known for the precipitation mechanism induced by TCA. For ammonium sulfate precipitation, the ions NH_4_^+^ and SO_4_^2−^ compete for water molecules and cause a disruption on the hydration layer around proteins molecules. As a result, aggregation and eventual precipitation take place [169,171].

Diverse examples of successful recovery of biopolymers by precipitation are found in the literature. Chen et al. [172] recovered pectic polysaccharides from fruit canning industry effluent by using five volume equivalents of ethanol to induce precipitation. For this technology to be economically feasible, the authors concluded that prior concentration (by four-fold) of the effluent is necessary. Patel et al. [167] recovered high molecular weight polysaccharides from culture broth of *Porphyridium cruentum*. By using isopropanol (2 volume equivalents), a recovery yield of 4.6 mg/L was achieved. Crowell et al. [173] showed that as long as the salt concentration is high enough (typically between 1 and 100 mM) and using acetone 80%, different types of water-soluble proteins can be precipitated in high yields (80%–100%).

One of the main drawbacks of solvent precipitation consists of the large volumes of solvent that are required. The required amounts of alcohol usually applied range from three to four in relation to the volume of polymer solution to be treated, which is equal to 75%–80% of the total volume [174]. Likewise, Marcati et al. [165] pointed out that precipitation usually results in an end-product incompatible with industrial application due to its low purity and poor solubility. Likewise, poor solubility in water was also a limitation found by other studies [167,171]. The lack of selectively has a direct implication on the operational costs as it will then require several sequential purification steps.

#### 6.2.2. Ultrafiltration

The concentration of biopolymers in wastewater is often too low to be economically feasible to concentrate it by evaporation or freeze-drying, while from the previous subsection, it may be concluded that also precipitation approaches require a large amount of chemicals. Membrane processes can be employed as a concentration method in the recovery of natural polymers, concentrating them in the retentate [175]. Polymers and inorganic compounds (alumina, titania, and zirconia oxides) are the traditional materials employed to fabricate membranes [175,176]. Most membranes processes use a pressure gradient as the driving force to separate molecules in solution, based on their molecular size and/or charge. Membrane separation processes are of great interest due to their high throughput, compactness, ability to work on a continuous regime, and because no chemicals are required to promote recovery [176,177].

In the context of recovery of biopolymers, ultrafiltration (UF) is most commonly used. Several examples of successful concentrations of biopolymer aqueous streams have been reported. Lo et al. [135] were able to retain nearly all protein present in poultry wastewater using UF. The recovered stream was 3.4 times more concentrated in comparison to the initial feed. Luo et al. [178] exploited a two-stage UF/NF process to recover whey protein from dairy wastewater. A total of 100% of the proteins were retained in the concentrate. The permeate was rich in lactose, another value-added molecule present in the dairy wastewater. NF was then used to concentrate the lactose steam by a factor of 5. Bahl et al. [168] recovered about 90% of polysaccharides present in a culture broth (1 g/L) of *Sphingomonas pituitose* by concentrating them five-fold. Similarly, Giacobbo et al. [179] concentrated (five-fold) polysaccharide and phenolic compounds from a clarified winery effluent by UF. These results were obtained by employing a polypropylene flat-sheet membrane with MWCO equal to 2 kDa. Other relevant studies are summarized in Table 5. We can thus conclude, based on the reviewed articles, that many commercial membranes can effectively concentrate biopolymer solutions.

The concentration of biopolymers by membrane processes requires careful design of the process in order to avoid a rapid decline of the membrane performance. Typically this is characterized by a reduction in permeate flux resulting from concentration polarization and fouling. These phenomena consist of the accumulation of solute on the membrane surface (concentration polarization), which can later adsorb on the surface and cause fouling. Besides reducing the permeate flux, the fouling layer can control membrane selectivity by acting as a secondary membrane. This may sometimes help, but when it is desired to avoid this issue, it is necessary to operate below a critical flux, which is the maximum flux in which the relationship between flux and transmembrane pressure is linear. This, however, implies a larger membrane area is required to maintain reasonable permeate flow, affecting the cost of the process [180].

Fouling can also be reduced by proper selection of the material of the membrane. Commercial UF membranes are often made of hydrophobic polymers, which are prone to fouling, especially caused by proteins [176,181]. Polysulphone and polyethersulphone are examples of materials relatively less hydrophobic due to the presence of sulfur dioxide units and, consequently, often proposed for the recovery of biopolymer, as shown in Table 5.

The driving force of ultrafiltration (pressure) can also be an issue as it can cause denaturation of proteins [135]. Alternatively, forward osmosis (FO), a concentration gradient-driven process, can be used. FO is able to concentrate biopolymer solutions due to the migration of water, through a membrane, from the feed solution side to the draw solution side. Another limitation of membrane processes is that they often lack selectivity, meaning that the final concentrate is still a mixture of biopolymers with different chemical structures. A few examples of fractionation of biopolymers by membrane processes have been reported in the literature. Marcati et al. [165] proposed a two-step ultrafiltration process to fractionate proteins from polysaccharides produced by *Porphyridium cruentum* (marine microalgae). The initial feed stream was composed of 40% polysaccharide (0.6 g/L) and 60% protein (0.9 g/L). After the second filtration stage, the polymer composition in the concentrate was 90% proteins, while the permeate mostly contained polysaccharides (80%) and a small fraction of protein. This enabled the recovery of proteins with a purity index of 2.3. In addition, Zhao et al. [182] fabricated membranes based on sulfonated polysulfone (s-PSf) and polyethylene glycol (PEG). s-PSf incorporation led to a negative membrane surface charge and still resulted in a poor fractionation of polysaccharides from protein. The initial stream was composed of polysaccharide/protein in equal mass, and after the filtration, the concentrate contained 1.25 times more polysaccharide than protein. The authors highlighted that this novel membrane showed a higher critical pressure (2.5 bar), when compared to commercial membranes, which demonstrates the antifouling property of the membrane.

**Table 5:** Examples of membrane processes reported in the literature, used for the concentration of biopolymers from different matrix.

Membrane Technology	Target Biopolymer	Matrix	Membrane Features	Key Findings	Ref.
**Ultrafiltration** **(diafiltration)**	Polysaccharide	Culture broth (*Porphyridium* *cruentum*)	Polyethersulfone Flat sheet MWCO: 300 kDa	64% of the polysaccharide recovered in the permeate Final purity in retentate fraction: 57% Purity 10-fold higher than initial	[167]
**Ultrafiltration**	Polysaccharide	Culture broth (*Sphingomonas* *pituitose*)	Polyethersulfone Hollow fiber MWCO: 55 kDa	Stream was concentrated by 5-fold 90% of polysaccharides in the retentate fraction	[168]
**Ultrafiltration**	Polysaccharide	Synthetic solution	Polysulfone Hollow fiber MWCO: 6 kDa	56% of arabinoxylan was in the retentate fraction, and stream was concentrated by 2-fold 76% of rhamnogalacturonan was in the retentate, and stream was concentrated by 5-fold	[183]
**Ultrafiltration**	Polysaccharide Protein	Culture broth (*Spirulina platensis*)	Polyether sulfone Flat sheet MWCO: 5 kDa	Concentration factor was 40-fold 0.2 g/L EPS solution was obtained in the retentate	[184]
**Ultrafiltration**	Protein	Poultry processing wastewater	Polysulfone Flat sheet MWCO: 30 kDa	Stream was concentrated by 3-fold 100% recovery of protein in the retentate Loss of protein in retentate due to fouling	[135]
**Ultrafiltration**	Protein	Poultry processing wastewater	Regenerated cellulose Spiral-wound MWCO: 30 kDa	Stream was concentrated by 7-fold concentration 100% recovery of protein in the retentate	[181]
**Ultrafiltration**	Protein	Fermentation broth (Ethanol production from corn)	Regenerated cellulose Flat sheet MWCO: 5 kDa	Concentration factor was 2-fold 80% of protein was in retentate 2-fold purification factor	[185]

#### 6.2.3. Aqueous Two-Phase Systems (ATPS)

**Figure 11:**
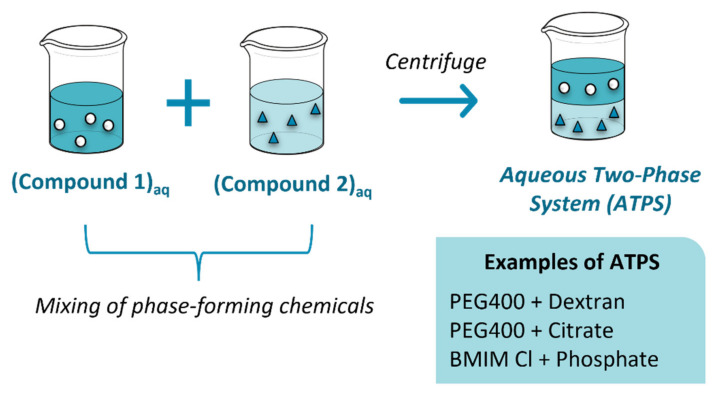
Schematic view of aqueous two-phase system formation.

Conventional liquid-liquid extraction, using organic solvent, is not suitable for many of the biopolymers produced from wastewater. These biopolymers usually have low solubility in these systems and, in particular, for proteins, conformation changes are another concern. A more suitable option is the application of aqueous two-phase systems (ATPS). ATPS is liquid-liquid extraction media based on two co-existing aqueous phases. ATPS is formed by mixing a pair of aqueous solutions above critical concentrations for phase splitting [186,187,188]. ATPS formation is displayed schematically in Figure 11. ATPSs can be formed by a pair of polymers [189,190,191], polymer/salt [192,193], polymer/ionic liquid [194,195], ionic liquid/salt [196,197,198] and short-chain alcohols/salt [199,200].

Aqueous two-phase systems have several benefits. The main advantage is that the systems are comprised mainly of water, which is an excellent solvent for biopolymers. Other advantages include minimal flammability, potentially high selectivity, and low energy requirement. This recovery technique is also able to concentrate the target compound by extracting them into the smaller volume of the extraction phase. Finally, similar to traditional liquid-liquid extraction, ATPSs are capable of working in a continuous regime, as shown in a recent literature review [201].

The number of publications demonstrating the high efficiency of ATPSs on recovering natural polymers from different sources is large, as summarized in Table 6. In the next subsection, the different types of ATPS will be briefly discussed. The mechanisms underlying the formation of two phases in each ATPS as well their key advantages will be presented. Finally, examples of recovery of natural polymers from diverse matrices by ATPS will be shown.

##### Polymer/Polymer ATPS

Polymer/polymer ATPS systems were the first to be investigated to recover natural macromolecules in an investigation carried out by Albertsson [202] in the early 1960s. This system is formed because of the intrinsic incompatibility between certain pairs of polymers. In other words, mixing aqueous solution of these two polymers forms aggregates and, eventually, phase splitting due to steric exclusion [203]. Polyethylene glycol (PEG)/dextran [189] and PEG/polyacrylates [191] and are examples of polymer/polymer systems often reported in the literature. This type of system is ideal for the separation of molecules that are sensitive to high ionic strength environments [204].

Badhwar et al. [205] applied PEG/dextran ATPS to purify polysaccharides produced by *A. pullulans*. The authors found that the biomass partitioned in the PEG-rich top phase and 90% of pullulan (the targeted polysaccharide) was present in the dextran-rich bottom phase. To recover the polysaccharide from the dextran-rich phase, acetone was used as an anti-solvent to induce precipitation. Compared to direct solvent precipitation, the proposed ATPS required lower chemical amounts, which significantly lowers the cost of the recovery process. Singh and Tavana [206] employed PEG/dextran ATPS to extract a model protein from an aqueous solution. Similar to the previous study, collagen (the targeted molecule) was mostly present in the dextran-rich bottom phase. It was also found that decreasing the molecular weight of the phase-forming polymers (PEG and dextran) increased the partitioning of collagen to the dextran-rich bottom phase. More examples of polymer/polymer ATPS for the recovery of biopolymers can be found in Table 6.

##### Polymer/Salt ATPS

The advantage of polymer/salt systems, when compared to polymer/polymer ones, is their lower costs for the phase-forming compounds, as well as a broader polarity range of the phases. The broader polarity range of the phases improves separation selectively. The most commonly used salts are phosphate, sulfate, and citrate [186,207]. The salting-out effect is responsible for the formation of this class of ATPS. In order words, the intense ion-dipole intermolecular interactions between salt and water molecules lead to steric exclusion of polymer. As a result, phase splitting takes place [204].

Diverse examples of successful polymer/salt systems for the recovery of biopolymers can be found in the literature. For example, Gatica et al. [208] employed a PEG/citrate ATPS to extract microbial, high molecular weight hyaluronic acid. This polysaccharide was mostly extracted in the citrate-rich bottom phase (80%), and it could be recovered with a purity level of 75%. The authors found that ATPS composed of higher molecular weight PEG improved the purity of the targeted biopolymer. The recovery of polysaccharides by polymer/salt ATPS was also investigated by Jiang et al. [209]. The separation of proteins from an exopolysaccharide produced by *L. actobacillus plantarum* was achieved using a PEG/phosphate salt. A total of 72% of the polysaccharide was found in the salt-rich phase, and no significant protein was found in the salt-rich phase. Ivyaswami et al. [210] investigated the recovery of proteins from fish industry effluent by PEG/citrate ATPS. More than 85% of the initial protein was in the PEG-rich phase. A similar trend was reported by Chow et al. [211], as, under optimal conditions, 90% of BSA is partitioned to the polymer-rich phase in PEG/phosphate ATPS. Besides high yields, the reported findings also demonstrated the selectivity of polymer/salt systems as different biopolymers were preferentially partitioned to opposite phases.

##### Alcohol/Salt ATPS

Alcohol/salt ATPS are generally composed of water-miscible alcohols (i.e., methanol, ethanol, and propanol). Similar to polymer/salt, ATPS, phosphate, sulfate, and citrate are the most commonly used salts as phase-forming compounds. The popularity of alcohol-based ATPS for the recovery of biopolymer is still limited, even though it has benefits when compared to its polymer-based counterparts. For instance, this type of ATPS is relatively inexpensive and characterized by lower viscosity and reduced settling times. Another advantage is that alcohol, one of the phase-forming compounds, can be easily recovered by evaporation [204].

Studies have demonstrated that alcohol/salt systems are effective as recovery techniques for different natural polymers from several natural sources. For instance, Li et al. [212] investigated the recovery of a glycoprotein (interferon α-2b) present in fermentation broth from recombinant *Escherichia coli* by alcohol-based ATPS. The alcohol-rich phase was the preferential phase as 75% of the targeted macromolecule was extracted to it. The purity was 16 times higher than in the crude stock. These results were achieved by using an ATPS composed of 2-propanol, ammonium sulfate and sodium chloride as additive. Cheong et al. [213] compared PEG1000/ammonium sulfate ATPS and ethanol/ammonium sulfate ATPS for the fractionation of polysaccharides and proteins. The polymer/salt system was not selective as both polysaccharides and proteins were extracted in large amounts to the salt phase (90% and 83%, respectively). The ethanol/ammonium system was much more selective, as most polysaccharides as extracted to the salt-rich phase (67%), while a lower extraction efficiency of protein to this phase was observed (18%). Based on the reviewed papers, it can then be concluded that in alcohol-salt systems, the preferential phase of polysaccharides is the salt-rich phase, while the proteins are mostly found in the alcohol phase.

##### Ionic Liquid (IL)-Based ATPS

Ionic liquids have recently emerged as replacements for salts and polymers in ATPS. This class of compound is defined as salts that are liquid at a significatively lower temperature (below 100 °C) compared to conventional salts [214,215]. The main advantage of using ionic liquids is that the large variety of ILs, possibly due to a large number of cation/anion combinations, allows proper control of the polarity of the phases [215]. As a result, systems with improved selectivity can be designed.

Also, this type of ATPS has been reported to be effective for the recovery of biopolymers. An IL-based ATPS was used by Du et al. [216] for direct extraction of protein from human urine. Potassium phosphate and 1-butyl-3-methylimidazolium chloride (BMIMCl) were the phase-forming compounds that were employed. By SDS-PAGE results, the authors showed that the majority of the proteins (molecular weight between 66 and 90 kDa) were present in the BMIMCl-rich phase, while no proteins were detected in the salt-rich phase. In addition, UV-Vis and FTIR spectroscopic showed that the proteins’ structure remained unchanged during the extraction process. Yan et al. [217] used an ATPS based on BMIMCl and phosphate salt to separate polysaccharides from protein in a mushroom crude extract. The authors found that 89% of polysaccharides were extracted in the phosphate-rich phase, while 88% of proteins were present in the IL-rich phase. The reviewed papers show that in ionic liquid/salt ATPS, polysaccharides are mostly present in the salt-rich bottom phase while protein is majorly in the ionic liquid-rich top phase. On the other hand, in ionic liquid/polymer ATPS, polysaccharides are generally extracted to the ionic liquid-rich bottom phase, while the polymer-rich top phase is the preferential phase of proteins. In both cases, the IL-rich phase was relatively more hydrophobic, and thus had a higher affinity for more hydrophobic molecules such as proteins.

##### Complex Coacervates

Similar to ATPSs, complex coacervate is an extraction platform formed by the two-coexisting aqueous phase. The main difference is that in complex coacervates, the phases are formed via associative phase splitting rather than segregative phase separation (as observed in ATPSs). Here, the biphasic system is formed by combining solutions of two oppositely charged macromolecules. After centrifugation, two phases are obtained: polyelectrolyte complexes and supernatant. As a recovery technique, the separation of molecules by complex coacervate occurs due to the partitioning of the target molecule between the coacervate-phase and liquid-phase [218]. Lindhoud et al. [219] demonstrated that lysozyme (protein) can be incorporated in a complex coacervate by mixing aqueous solutions of polyacrylic acid (negatively charged), poly-(N,N,dimethylaminoethylmethacrylate), and lysosome. It was possible to increase the concentration of protein from 6.5 to 150 g/L. The recovery of lysozyme from the polyelectrolyte complexes can be performed by changing the ionic strength of the media. By doing so, the complex coacervate is destabilized, and further purification is still required to obtain pure lysozyme. More investigations still need to be carried out, in particular concerning the selectivity of this technique.

#### 6.2.4. Back-Extraction and Recycling of Compounds

The possibility to back-extract the target molecule(s) and to recycle its phase-forming compounds is often under investigation. Yet, recycling and reusing of phase-forming compounds are essential to make ATPS a feasible technology for biopolymer recovery. In particular, recovery of ILs used in ATPS is highly desired as losses of ILs have a large impact on the process economy, and maintaining them in the process can effectively minimize environmental impacts and operational costs. In the ATPS context, the work of Claudio et al. [220] is one of the few examples addressing back-extraction and recycling. The authors proposed a recovery process of gallic acid, present in synthetic solution, based on two ionic liquid(IL)/salt ATPS. The first step ATPS, composed of sodium sulfate and BMIM dicyanamide, extracted more than 90% of the target molecule into the IL-rich phase. To recover the desired compound from the IL-rich phase after the extraction, sodium carbonate (about 10 wt%) was added to the IL-rich phase loaded with gallic acid. Formation of a new ATPS occurred, in which 72% of gallic acid was back-extracted into the Na_2_CO_3_-rich phase, allowing the regeneration of the ionic liquid. The regenerated IL-phase was used in a new extraction cycle, and the extractive performance was maintained. Suarez-Ruiz et al. [221] proposed the recovery of proteins and recycling of ionic liquid by ultrafiltration. It was possible to recover 96% of IL and 82% of protein using a centrifugal filtration device. The relatively low recovery of IL (96% is truly unacceptable on an industrial scale, especially not when considering that the recovered protein is then only a fraction of the amount of lost IL) was explained by the inefficient design of the experiments as the scope of the paper was on proof of concept rather than on optimizing the operational conditions of filtration. Li et al. [222] also carried out an investigation recycling phase-forming compounds. The authors found that by using a polypropylene glycol (PPG)/cholinium propionate ATPS, nearly 100% of proteins were extracted to the PPG-rich phase. Subsequently, the regeneration of the PPG-rich phase was accomplished by increasing the temperature of the PPG-rich phase up to 45 °C. As a result, 90% of the polymer was recovered. Recoveries such as this, losing 10% of the phase-forming agent, are, in our opinion far below recovery rates that are necessary for industrial operation to be sustainable and economical. Therefore, more research should be directed toward the higher recovery of phase-forming agents, possibly through a combination of steps, of which the reported temperature swing [222] could be a first step, followed by a polishing step. Based on the difference in molar weight of the large protein and the much smaller PPG applied (PPG_400_), membrane-based fractionation could also be feasible here to fully recover the PPG and reuse it.

**Table 6:** Examples of ATPS, reported in the literature, used for the recovery of biopolymers from different matrices.

ATPS	Target	Composition wt%	Matrix	Key Findings	Ref.
Polymer/polymer	Protein (amyloglucosidase)	PEG 4000: 13% NaPA 15000: 11%	Synthetic solution	86% of the enzyme was present in PEG-rich phase Improvement on purity by a factor of 2	[223]
Polymer/polymer	Protein (interferon α-2b)	PEG 600: 30% PPG 400: 30%	Culture broth *(Escherichia coli)*	90% of the protein in the PEG-rich phase Improvement on purity by a factor of 2	[224]
Polymer/salt	Protein (Rubisco)	PEG 400: 39% Citrate: 25%	Synthetic solution	97% of rubisco is extracted to PEG-rich phase Protein kept native form after extraction	[207]
Polymer/salt	Protein (β-lactoglobulin α-lactalbumin)	PEG 1500: 14% (NH_4_)_2_SO_4_: 26%	Cheese whey	95% of protein recovered as precipitate Purity equal to 80%	[225]
Polymer/salt	Polysaccharide (mannose, glucose, galactose units)	PEG 600: 23% NaH_2_PO_4_: 17%	Culture broth (EPS from *Lactobacillus* *plantarum*)	72% of polysaccharides in salt-rich phase No protein was observed in salt-rich phase	[209]
Alcohol/salt	Protein (recombinant green fluorescence protein)	1-PrOH: 33% Citrate: 18%	Culture broth (*Escherichia coli*)	92% of protein is in citrate-rich phase	[226]
Alcohol/salt	Protein (pectinase)	EtOH: 19% K_3_PO_4_: 22%	Crude extract from Mango wastes	97% of pectinase was extracted to ethanol-rich phase, and purity increased by a factor of 11	[227]
Alcohol/salt	Polysaccharide	EtOH: 15% Na_2_CO_3_: 20%	Crude extract from *Cordyceps sinensis*	97% of polysaccharide extracted to salt-rich phase Purity > 70%	[228]
IL/salt	Polysaccharide protein	EMIMCl: 15% K_3_PO_4_: 22%	Crude extract from *Isochrysis galbana* (microalgae)	100% of the protein extracted to IL-rich phase 61% of the polysaccharides in salt-rich phase	[229]
IL/salt	Protein (BSA)	C_8_MIMCl:21% K_2_HPO_4_: 28%	Synthetic solution	100% of BSA is extracted into IL-rich phase Protein kept native form after extraction	[230]
IL/salt	Polysaccharide protein	BMIMCl:16% K_3_PO_4_: 22%	Fermentation broth (*Cordyceps sinensis*)	89% of polysaccharides in salt-rich phase 88% of proteins extracted into IL-rich phase	[217]
IL/polymer	Protein (amyloglucosidase)	PPG_400_: 30% [Ch][DHCit]: 30%	Fetal bovine serum matrix	100% extraction of protein to PPG-rich phase, even at high protein concentrations (10 g/L) Protein kept native form after extraction	[231]
IL/polymer	Protein (interferon α-2b)	PEG_400_: 37% [Ch][DHP]: 36%	Synthetic solution	80% of rubisco is extracted to PEG-rich phase Protein kept native form after extraction	[207]

### 6.3. Three-Phase Partitioning Systems (TPPs)

Three-phase partitioning systems combine salting-out phenomena and solvent precipitation to concentrate and fractionate polysaccharides and proteins. TPPs can also be understood as a particular case of an aqueous two-phase system. TPPs form by mixing t-butanol, inorganic salt (typically ammonium sulfate), and an aqueous solution of biopolymers as the target compounds. The presence of butanol and inorganic salts reduces the solubility of the biopolymers in solution and, consequently, induces the formation of a third phase at the interface. The accumulation of the biopolymer at the interface promotes concentration and makes it easier to recover the target molecule [232,233].

Some examples of recovery of natural polymers by three-phase partitioning systems have been reported. Wang et al. [234] proposed the use of TPPS for separating the polysaccharide fraction present in the fermentation broth of *Phellinus baumii*. The system was formed by dimethyl carbonate as organic phase and sodium citrate (19% *w/v*) as salt phase. The TPP was able to extract more than 60% of polysaccharides to the citrate phase. This technique was also selective, as 90% of biopolymer present in the citrate phase was polysaccharide. Sharma and Gupta [235] showed that 70% of alginate (polysaccharide) accumulated in the interfacial precipitate when using a TPP based on ammonium sulfate (13 wt%) and t-butanol (14 wt%). Belchior and Freire [236] fractionated the different types of proteins found in egg white. To do so, the authors employed a combined approach based on ATPS and TPP. The system was composed of PEG_2000_ (30 wt%) and phosphate salts (13 wt%). It was observed that 82% of ovalbumin accumulated in the PEG-rich phase, while 77% of lysozyme was in the interfacial precipitate. High purity of ovalbumin in the PEG-rich phase was obtained, while lysozyme still required further purification steps as other egg proteins were also present in precipitate.

### 6.4. Final Considerations on EPS Recovery

When it comes to the recovery of biopolymers, fractionation and concentration are the main steps involved in the process. These steps can be carried out by different techniques. For instance, solvent precipitation is a relatively easy concentration technique and is already applied on an industrial scale. However, this technique leads to dilute solutions and requires large volumes of organic solvent. Another drawback is the relatively low purity of the end product. Alternatively, membrane processes can effectively concentrate biopolymer solutions. To do so, a proper design of the process (in terms of membrane material, molecular weight cut-off, and transmembrane pressure) has to be thoroughly performed in order to avoid the rapid decline of the membrane performance. For fractionation of biopolymers, an aqueous two-phase system can be regarded as a promising technology. The reviewed papers have been shown that the different types of ATPSs result in different yields and recovery of different types of biopolymer from aqueous streams. Therefore, the choice of the appropriate phase-forming compounds needs to consider the desired characteristics of the end product in terms of purity, yield, and nature of the biopolymer.

Yet, some aspects of ATPS still require deeper investigation. For instance, the recycling of phase-forming compounds in ATPS is absolutely necessary for the process feasibility, as this can significantly reduce the operational costs and environmental impact. Following this, the partition behavior involved in ATPS is still not well understood. Consequently, more knowledge needs to be gathered so that the current predictive models can be further developed. Other challenges of this recovery technique have to do with phase separation. For example, some systems have low interfacial tension, which makes the phase separation relatively more difficult due to slow coalescence. In the same way, the small density differences also negatively affect phase settling under the influence of gravity. These aspects consequently hinder its application in a continuous regime, which can possibly explain why ATPSs have not yet been incorporated by the industry even though they have been studied over the past 70 years. From the environmental point of view, most studies apply phosphate and ammonium salts as one of the phase-forming compounds, and inappropriate disposal of wastewater rich in those salts can cause eutrophication in aquatic systems. As an alternative, biodegradable salts, such as citrates, should be further investigated as a replacement of phosphate/ammonium salts in order to confirm their effectiveness in forming an ATPS and promoting a selective separation. To conclude, a combination of ATPS and membrane processes represent a potential process to fractionate and concentrate biopolymers from aqueous streams.

## 7. Conclusions

In this review, recovery of acidic species from fermented wastewater and fractionation of the acidic species from other species present in the broth have been discussed. For the smaller acids, the volatile fatty acids, adsorption appears to be the approach to obtain the acids in their acid form, while ion exchange has the benefit that it can be applied at higher pH, but the limitation that extra conversion from the carboxylate is needed to obtain the acids. This may be performed through CO_2_-expanded alcohols as solvents. For the larger medium-chain carboxylic acids and long-chain dicarboxylic acids, the limited solubility in water offers opportunities during recovery. Through electrochemical pH change, oiling out can be practiced, as the neutral acids are much less soluble in water than their carboxylate equivalents that are formed under fermentation conditions. The electrolysis can even be combined with pertraction and/or electrodialysis to facilitate the concentration step needed to reach supersaturated concentration. As the fourth category, unsaturated fatty acids to be obtained by pyrolysis of polyhydroxyalkanoates have been discussed, and for these acids, there is hardly any information yet about purification strategies. Based on their close boiling behavior, separation of C_4_ and C_5_ unsaturated acids by distillation might be difficult, while the one carbon atom difference might offer opportunities for fractionation by liquid extraction. The last category in this review was the extracellular polymeric substances, of which the acid-bearing alginate is an important polymer. Fractionation of this polymer from others has been discussed, and the use of ATPS for fractionation appears most interesting, while obtaining mixtures of EPS also appears well-possible using membrane approaches for concentration, followed by a precipitation step.

## Figures and Tables

**Table 1:** Adsorption characteristics and possible interactions between carboxylic acids (HA)/carboxylate (A^−^) and amine base (B)/ammonium compounds (Q). X^−^ is an inorganic anion. Reproduced with permission from [55], published by Elsevier, 2014.

Fermentation pH	Main Carboxylate Species	Amine B or Ammonium Compound Q	Interaction Mechanism	Remarks
**pH < pK_a_**	HA	Primary, secondary, or tertiary amine (B)	• Acid-base reaction leading to BH + A^−^ ion pairing • H-bonding B:HA	Ion pairing prevails over H-bonding for strong basic B H-bonding between the amine and hydroxyl moiety of the acid
		Primary, secondary, or tertiary ammonium salt (QH + X^−^)	• H-bonding QH + X^−^:HA • Anion exchange reaction leading to QH + A^−^	Anion exchange will occur only for A^−^ being weaker base than X^−^
		Quaternary ammonium hydroxide (Q + OH-)	• Anion exchange reaction leading to Q + A^−^	
		Quaternary ammonium salt (Q + X^−^)	• H-bonding Q + X^−^:HA • Anion exchange Reaction leading to Q + A^−^	Anion exchange will occur only for A^−^ being weaker base than X^−^
**pH < pK_a_**	A^−^	Primary or secondary amine (B)	• H-bonding B:A	H-bonding between the amine and carbonyl group of the acid
		Primary, secondary, or tertiary ammonium salt (QH + X^−^)	• Anion exchange reaction leading to QH + A^−^	
		Quaternary ammonium hydroxide (Q + OH-)	• Anion exchange reaction leading to Q + A^−^	
		Quaternary ammonium salt (Q + X^−^)	anion exchange reaction leading to Q + A^−^	

**Table 2:** General comparison between the desorption techniques applied for various adsorbents in recovering carboxylic acid from a fermentation broth.

Adsorbent	Functional Group	Adsorbent Regeneration Technique	Remarks	Limitations	Ref.
Activated carbon		Basified organic solvents as eluent	Carboxylates are recovered. Energy duty not in this stage, but with regeneration from the basified solvent	Requires extra distillation step to recover the organic solvent and carboxylate salts from the eluent Recovers the acids as a carboxylate salt	[50]
Synthetic polyaromatic resins	Non	Thermal desorption	Recover the VFAs in their acid form Enables the fractionation of the acids High concentration factor can be achieved	Energy demand in regeneration can be high, depending on the acid to water ratio in the adsorbent pores	[28]
Functionalized synthetic polyaromatic resins	Pyridine, imidazole, and primary, secondary or tertiary amine (weak base)	Base eluents Mineral acid eluents	Carboxylates are recovered. Energy duty not in this stage, but with regeneration from the basified solvent	Stochiometric waste salt coproduction Requires extra agent to protonate the carboxylates Requires extra separation method to recover the acids as a carboxylate salt from the eluent	[55]
		Organic solvents as eluent		Requires extra step to recover carboxylate salts from the eluent	
Functionalized synthetic polyaromatic resins	Quaternary ammonium (strong base)	Base eluents		Stochiometric waste salt coproduction when carboxylates are targeted Requires extra step to recover carboxylate salts from the eluent	[55]
Functionalized synthetic polyaromatic resins	Quaternary ammonium (strong base)	CO_2_ expanded alcohol	No stochiometric waste salt production Combined desorption and esterification of the acids	High pressure required for acid desorption Requires extra step to recover carboxylic acids from the eluent (e.g., distillation)	[56]

**Table 3:** Overview of reported pyrolysis results for PHB(V) toward crotonic acid.

HB Content PHBV (mol%)	Catalyst	Degradation Temperature (°C)	Pyrolyis Temperature (°C)	Crotonic Acid Yield (wt%)	Pyrolyzates Composition (wt%)	Ref.
100	No	260–290	260	N/A	Crotonic acid 67.7 Isocrotonic acid 3.1 Oligomers 29.2	[13]
100	No	290	290	62.5	Crotonic acid 63.8 Isocrotonic acid 1.0 Oligomers 33.7 3-hydroxybutyric acid 1.5	[16]
88	No	280–290	280	N/A	Crotonic acid 60.34 2-pentenoic acid 7.13 Oligomers 32.53%	[119]
88	Yes (MgOH_2_)	240–250	260	N/A	Crotonic acid 85.31 2-pentenoic acid 10.92 Oligomers 3.77	[119]
100	No	280	280	57.0	Crotonic acid 57.1 Isocrotonic acid 3.6 Oligomers 39.3	[14]
100	Yes (MgOH_2_)	240	240	83.0	Crotonic acid 97.7 Isocrotonic acid 0.6 Oligomers 1.7	[14]
98.95	No	280–290	290	N/A	Crotonic acid 58.09 2-pentenoic acid 0.51 Isopropyl-2-crotonic acid 38.38 Butyric-2-crotonic acid 2.9	[15]
100	No	300–310	310	65	Crotonic acid 57.1 Isocrotonic acid 5.0 Oligomers 37.9	[84]
100	No, NaOH pretreament	300–310	310	80	Crotonic acid 86.6 Isocrotonic acid 1.9 Oligomers 11.5	[84]
100	No	N/A	250	N/A	Crotonic acid 64.4 Oligomer 8.4	[116]
100	No	290	170 at 150 mbar	58	92% Crotonic acid	[107]

**Table 4:** Properties of crotonic acid and 2-pentenoic acid.

Property	Crotonic Acid	2-Pentenoic Acid
Molecular weight (g mol^−1^)	86.0892 [124]	100.117 [125]
Boiling point (°C @ 760 mmHg)	184.7 [124]	200–203 [126]
Melting point (°C)	72 [124]	8–10 [126]
Water solubility (g L^−1^ @ 25 °C)	94 [124]	62.9 [125]
Density (g Ml^−1^ @ 25 °C)	1.027 [127]	0.99 [128]
pK_a_ (@ 25 °C)	4.817 [124]	5.02 [129]

## Data Availability

No new data have been generated. All reported data are obtained from previously published articles and are accessible.
